# Genome assembly provides insights into the genome evolution and flowering regulation of orchardgrass

**DOI:** 10.1111/pbi.13205

**Published:** 2019-07-30

**Authors:** Linkai Huang, Guangyan Feng, Haidong Yan, Zhongren Zhang, Bradley Shaun Bushman, Jianping Wang, Aureliano Bombarely, Mingzhou Li, Zhongfu Yang, Gang Nie, Wengang Xie, Lei Xu, Peilin Chen, Xinxin Zhao, Wenkai Jiang, Xinquan Zhang

**Affiliations:** ^1^ Department of Grassland Science Animal Science and Technology College Sichuan Agricultural University Chengdu China; ^2^ School of Plant and Environmental Sciences Virginia Tech Blacksburg VA USA; ^3^ Novogene Bioinformatics Institute Beijing China; ^4^ Forage and Range Research Laboratory USDA‐ARS Logan UT USA; ^5^ Agronomy Department University of Florida Gainesville FL USA; ^6^ Animal Science and Technology College Sichuan Agricultural University Chengdu China; ^7^ State Key Laboratory of Grassland Agro‐Ecosystems College of Pastoral Agriculture Science and Technology Lanzhou University Lanzhou China

**Keywords:** *Dactylis glomerata*, reference genome, long‐read sequencing, transposon, flowering time

## Abstract

Orchardgrass (*Dactylis glomerata* L.) is an important forage grass for cultivating livestock worldwide. Here, we report an ~1.84‐Gb chromosome‐scale diploid genome assembly of orchardgrass, with a contig N50 of 0.93 Mb, a scaffold N50 of 6.08 Mb and a super‐scaffold N50 of 252.52 Mb, which is the first chromosome‐scale assembled genome of a cool‐season forage grass. The genome includes 40 088 protein‐coding genes, and 69% of the assembled sequences are transposable elements, with long terminal repeats (LTRs) being the most abundant. The LTRretrotransposons may have been activated and expanded in the grass genome in response to environmental changes during the Pleistocene between 0 and 1 million years ago. Phylogenetic analysis reveals that orchardgrass diverged after rice but before three Triticeae species, and evolutionarily conserved chromosomes were detected by analysing ancient chromosome rearrangements in these grass species. We also resequenced the whole genome of 76 orchardgrass accessions and found that germplasm from Northern Europe and East Asia clustered together, likely due to the exchange of plants along the ‘Silk Road’ or other ancient trade routes connecting the East and West. Last, a combined transcriptome, quantitative genetic and bulk segregant analysis provided insights into the genetic network regulating flowering time in orchardgrass and revealed four main candidate genes controlling this trait. This chromosome‐scale genome and the online database of orchardgrass developed here will facilitate the discovery of genes controlling agronomically important traits, stimulate genetic improvement of and functional genetic research on orchardgrass and provide comparative genetic resources for other forage grasses.

## Introduction

Grasslands are an essential global resource for grazing and improving the environment and occupy over 25% of the land area of Earth (Afkhami *et al*., 2014; Jones and Pašakinskienė, 2005; Shantz, 1954). Forage grasses are the most important constructive component of grasslands (Barnes *et al*., 1995). Orchardgrass (*Dactylis glomerata* L.) belongs to Pooideae in the Poaceae family and is one of the most important cool‐season forage grasses cultivated worldwide. Indigenous to Eurasia and northern Africa, orchardgrass has been naturalized on nearly every continent and utilized as a pasture or hay grass (Hirata *et al*., 2011; Stewart and Ellison, 2010; Xie *et al*., 2015). As one of the top four economically important perennial forage grasses cultivated worldwide, orchardgrass is important for the production of forage‐based meat and dairy throughout the temperate regions of the world (Wilkins and Humphreys, 2003). Orchardgrass is particularly attractive for these conditions because of its high biomass yields, high carbohydrate levels, shade tolerance and adaptability to abiotic stress (AnneMarteTronsmo, 1993; Turner *et al*., 2007; Volaire, 2003; Volaire *et al*., 2001). Heading date is a surrogate measure for flowering time and is strongly correlated with the yield and quality of forage grasses. Due to the widespread geographical distribution of orchardgrass, its heading date is quite variable, which makes it ideal for studying how flowering time is regulated (Bushman *et al*., 2012; Sheldrick *et al*., 1986).

In contrast to most other major crops, forage grasses are subjected to multiple harvests per growing season for herbage yield rather than a single harvest for grain yield, and they harbour extensive variation and valuable abiotic/biotic stress resistance genetic resources for crop improvement due to their good adaptability to the natural environment (Bertrand *et al*., 2010; Moore *et al*., 1962; Talukder and Saha, 2017). Molecular breeding is an important approach in improving the breeding efficiency of forage grasses, but advancements in this field are hampered by limited genetic resources (Moose and Mumm, 2008; Ribaut *et al*., 2010). Acquiring a high‐quality reference genome for orchardgrass is paramount to strengthening the capabilities of molecular breeding and further promoting forage grass genetic and genomewide studies (Badouin *et al*., 2017; Brozynska *et al*., 2016; Huang *et al*., 2015; Nogué *et al*., 2016; Schulman *et al*., 2017; Varshney *et al*., 2014; Yan *et al*., 2016). *De novo* assemblies of cool‐season forage grasses have been limited by their large genome sizes (2–6Gb) with different ploidy levels (2–8×), high heterozygosity and high repetitive sequence content (Hegde *et al*., 2000; Kawube *et al*., 2015). Currently, the only forage grass with an available and appreciable reference genome is perennial ryegrass (*Lolium perenne* L.), which was sequenced using a second‐generation sequencing platform. However, its assembly quality (contig N50 = 16.37 kb; scaffold N50 = 70.06 kb) has limited its applications in functional genetic research on the species as well as on other forage grass species (Byrne *et al*., 2016).

Here, we report an assembly of the first chromosome‐scale reference genome of diploid orchardgrass, representing the first publicly available genome assembly in a cool‐season (C3) forage grass. Combining PacBio single‐molecule real‐time (SMRT) sequencing (Roberts *et al*., 2013), Hi‐C chromosome‐scale scaffolding, BioNano, 10× Genomics and Illumina short‐read sequencing (Belton *et al*., 2012; Mascher *et al*., 2017), we show an orchardgrass reference genome of 1.84 Gb with a contig N50 of 0.93 Mb, a scaffold N50 of 6.08 Mb and a super‐scaffold N50 of 252.52 Mb. Phylogenetic analysis reveals a common ancestor before ~17.5–27.6 million years ago (Mya) between orchardgrass and three Triticeae species. One evolutionarily conserved chromosome was detected by analysing chromosome derivation in these grass species. A total of 76 orchardgrass germplasm accessions with different origins were resequenced to understand their population structure and genetic diversity. Their flowering mechanisms were analysed, and several key candidate genes in orchardgrass were identified by an integrative approach combining quantitative genetics, gene expression analysis, quantitative trait locus (QTL) analysis and bulked segregant analysis (BSA). Additionally, an online database for the orchardgrass reference genome with integrated annotations, gene blast results and transcriptomic data has been developed (https://orchardgrassgenome.sicau.edu.cn). The results of this study provide a chromosome‐level reference genome assembly, an important resource with which to advance biological discovery and breeding efforts in orchardgrass, as well as comparative genetic resources for other forage grasses.

## Results

### Genome assembly, quality validation and annotation

The genome of an orchardgrass genotype, ‘2006‐1’, was initially sequenced using the Illumina, 10× Genomics and PacBio platforms to generate the V1.0 assembly. This assembly comprised 1.78 Gb of sequences, with a contig N50 of 1.05 Mb and a scaffold N50 of 3.41 Mb, accounting for 91.75% of the estimated genome size (Table 1; Tables S1 and S2; Figures S1 and S2). Of the 1.78Gb of scaffold sequences, 1.67 Gb (93.82%) was anchored to seven super‐scaffolds (chromosomes) using the Hi‐C platform (Figure S3; Tables S3 and S4; Figures S4 and S5; Appendix S1). The assembly was then elongated using BioNano to generate the V1.1 assembly with a contig N50 of 0.93 Mb and a scaffold N50 of 6.08 Mb, accounting for 94.84% (1.84/1.94) of the genome size. The chromosome anchoring to the seven super‐scaffolds was increased to 1.77 Gb (96.21%) by Hi‐C assembly.

**Table 1:** Statistics of the orchardgrass genome assembly

Genome assembly	v1.0		v1.1		
	Illumina + 10 × Genomics+PacBio		Illumina + 10 × Genomics + PacBio + BioNano		
	Contigs	Scaffolds	Contigs	Scaffolds	Super‐scaffolds
N50 (size/number)	1.05 Mb/513	3.41 Mb/132	0.93 Mb/574	6.08 Mb/92	252.52 Mb/4
N90 (size/number)	276.47 kb/1734	748.72 kb/559	238.95 kb/1980	1541.67 kb/310	213.52 Mb/7
Largest	7.70 Mb	32.90 Mb	7.70 Mb	22.88 Mb	276.68 Mb
Total size	1.76 Gb	1.78 Gb	1.78 Gb	1.84 Gb	1.84 Gb
Total number	4024	2045	5002	2110	1737

The completeness and base accuracy of the assembled orchardgrass genome were validated using BUSCO (Simão *et al*., 2017) and CEGMA (Parra *et al*., 2007). BUSCO showed that 96.7% of the 1440 single‐copy plant orthologues were complete, and CEGMA showed that the assembled genome completely covered 231 (93.15%) of the 248 core eukaryotic genes (CEGs) and partially covered 13 of the CEGs. Less than 2% of the CEGs were not detected (Table S5). The draft assembly was further evaluated by mapping short high‐quality reads to the genome assembly. The mapping rate was 99.62%, and the genome coverage was 99.66% (Table S6). A total of 53 836 publicly available expressed sequence tag (EST) sequences of *D. glomerata* were mapped to the genome with an identity >95%, and 49,017 (91.05%) of the sequences were mapped to the reference genome with more than 90% coverage (Table S7) (Bushman *et al*., 2011). High consistency between the Hi‐C and BioNano results was also observed, suggesting a reliable assembly (Figure S6). Collectively, these data indicated the high genome coverage of the orchardgrass assembly sequence.

A total of 40 088 protein‐coding genes were identified, 91% of which had functional annotations and 32 577 (81.26%) of which had evidence of transcription (Tables S3 and S8–S11). We also identified 799 transfer RNAs, 17510 miRNAs, 633 small nuclear RNAs and 400 ribosomal RNAs (Table S12). The orchardgrass reference genome with integrated annotations, gene blast results and transcriptomic data has been uploaded to an online database (https://orchardgrassgenome.sicau.edu.cn/).

### Evolution of transposable elements

**Figure 1:**
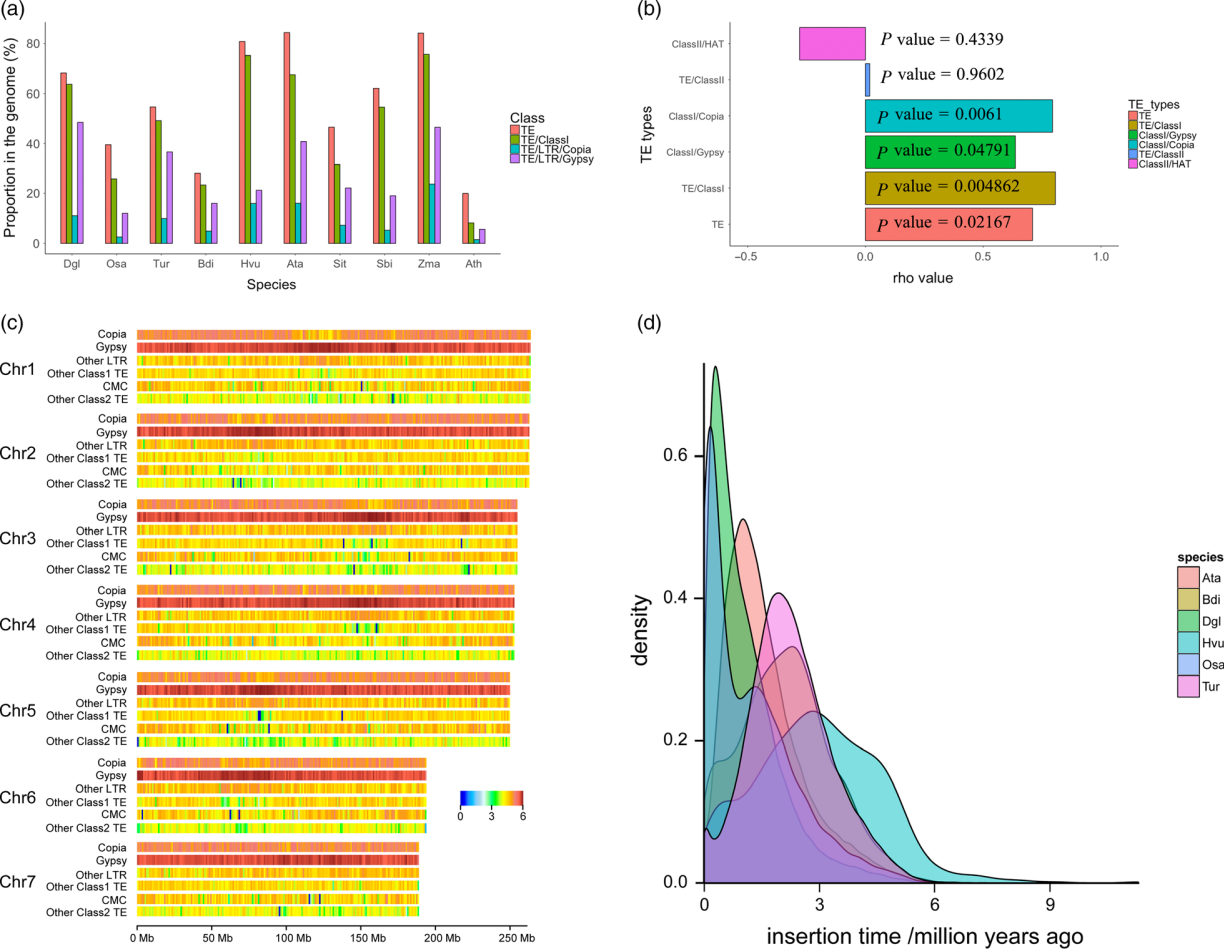
Characterization of transposons in orchardgrass. (a) Proportion of TEs (class I; LTR/Copia; LTR/Gypsy) in the genomes of Dgl (*D. glomerata*), Osa (*O. sativa*), Tur (*T. urartu*), Bdi (*B. distachyon*), Hvu (*H. vulgare*), Ata (*A. tauschii*), Sit (*S. italica*), Sbi (*S. bicolor*), Zma (*Z. mays*) and Ath (*A. thaliana*). (b) Spearman correlation analysis between plant genome size and proportion of TEs in the genomes of eight Poaceae species, *Arabidopsis* and orchardgrass. A rho value > 0 indicates a positive correlation; a rho value < 0 indicates a negative correlation. Very weak or no correlation: |rho*| < *0.2; weak: 0.2 ≤ |rho| < 0.4; moderate: 0.4 ≤ |rho| < 0.6; strong: 0.6 ≤ |rho| < 0.8; and very strong: 0.8 ≤ |rho| < 1. (c) Heatmaps of log of TE density along the seven chromosomes for Copia, Gypsy, other LTRs, other class I TEs, CMC and other class IITEs. (d) Insertion time of LTRs in six species, namely Ata, Bdi, Dgl, Hvu, Osa and Tur.

In total, 68.56% of the assembled genome sequences were annotated as transposable elements (TEs), 63.64% of which were retrotransposons and 4.92% of which were DNA transposons (Table S13). Of the retrotransposons, long terminal repeats (LTRs) constituted the vast majority, accounting for 61.15% of the genome (96% of the LTRs). Gypsy and Copia were the two major LTR superfamilies, and the proportion of Gypsy LTRs (48.36%) was higher in orchardgrass than in eight other Poaceae species and *Arabidopsis* (Gordon *et al*., 2017; Initiative, 2000; Ling *et al*., 2018; Luo *et al*., 2017; Mascher *et al*., 2017; Paterson *et al*., 2009; Schnable *et al*., 2009; Yu *et al*., 2002; Zhang *et al*., 2012; Table 1 and Tables S13 and S14; Figure 1a). Similarly, compared to the other species, orchardgrass contained larger proportions of subfamilies Gypsy/Athila (9.32%) and Copia/Sire (2.06%) (Table S15). Similar to the other species, orchardgrass contained LTR/TEs and DNA/TEs mainly distributed in gene flanking regions (3kb) (Figure S7). The density of Gypsy family LTRs increased from the telomere to the centromere, while the Copia family was uniformly distributed along the seven chromosomes (Figure 1c). In an analysis including eight Poaceae species, *Arabidopsis* and orchardgrass, we found a strong correlation between genome size and the proportion of TEs that were Gypsy and Copia LTRs (Figure 1b). These two LTR families were predicted to be amplified 0–1.0 million years ago (Mya) in the orchardgrass genome (Figure 1d), and the amplification of LTR/Copia appeared to have happened before the amplification of LTR/Gypsy (Figure S8), which may have led to the large genome size of orchardgrass.

The LTR amplifications were estimated to have taken place during the Pleistocene epoch, including the most recent ice age, lasting from 2.58 Mya until 10 000 years ago (Figure 1d; Figure S8). During the Pleistocene epoch, freezing weather and limited global atmospheric CO_2_ (180ppm) negatively impacted the growth of grasslands and other types of vegetation (Cerling, 1999). To survive during that time, most plants had to adapt to stressful abiotic conditions. As TEs become activated under stress, environmental stress likely led to the reorganization of plant genomes during this time period (Grandbastien, 1998), potentially facilitating adaptation to stressful environments in these species (Lisch, 2013; McClintock, 1983). We modelled the age of LTRs in six Poaceae species and found that the expansion of LTRs occurred earlier in orchardgrass than in rice but later than in*Brachypodium distachyon* and three Triticeae species, namely *Hordeum vulgare* (barley)*, Triticum urartu* and *Aegilops tauschii* (Figure 1d). Interestingly, the peak in LTR insertions corresponded to the order of species divergence, where orchardgrass diverged after rice from its common ancestor but before the three Triticeae species (Chen and Craven, 2007). Collectively, the LTR content and expansion time corresponded to the genome size and divergence time of grass species, suggesting that LTRs are involved in grass speciation.

### Phylogenetic evolution, genome synteny and chromosome derivation

**Figure 2:**
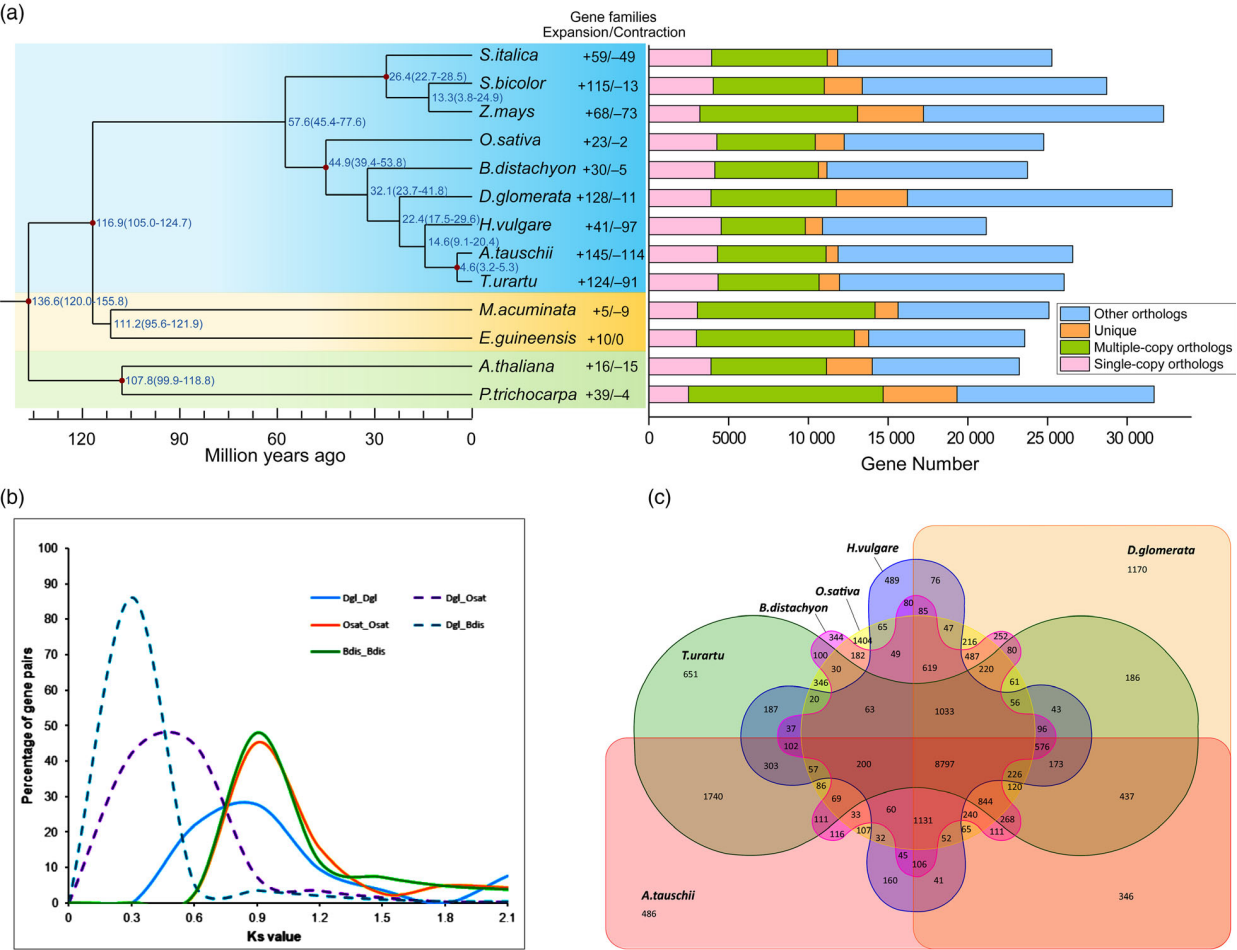
Gene family and genome evolution of orchardgrass. (a) The left panel includes the estimation of divergence time of orchardgrass and *O. sativa*,* T. urartu*,* B. distachyon*,* H. vulgare*,* A. tauschii*,* S. italica*,* S. bicolor*,* Z. mays*,* A. thaliana*,* P. trichocarpa*,* E. guineensis* and *M. acuminata*. The right panel displays the distribution of single‐copy, multiple‐copy, unique and other orthologues. (b) The number of gene families shared among six Poaceae species shown in Venn diagrams. Orchardgrass shares 8797 gene families with five other species, and 1170 gene families were unique to orchardgrass. (c) Distribution of the Ks values of the best reciprocal BLASTP hits in the genomes of *D. glomerata* (Dgl), *B. distachyon* (Bdis) and *O. sativa* (Osat).

Using the available genome resources, a unique set of gene families among 13 plant species, including orchardgrass and eight related grass species, were identified (D'hont *et al*., 2012; Gordon *et al*., 2017; Initiative, 2000; Ling *et al*., 2018; Luo *et al*., 2017; Mascher *et al*., 2017; Paterson *et al*., 2009; Schnable *et al*., 2009; Singh *et al*., 2013; Tuskan *et al*., 2006; Yu *et al*., 2002; Zhang *et al*., 2018). All species included in the analysis contained 33 981 gene families and shared 803 single‐copy and 596 multiple‐copy putative orthologous genes (Figure 2a). Orchardgrass and its closely relatives in Poaceae (*B. distachyon*,* H. vulgare*,* T. urartu*,* Oryza sativa* (rice) and *A. tauschii*) were clustered into one monophyletic group. These results suggest that orchardgrass diverged after riceand *B. distachyon* but before the three Triticeae species (Figure 2a). This phylogenetic tree is consistent with the species relationships observed in previous studies (Chen and Craven, 2007).

The orchardgrass genome size, LTR insertion peak and divergence times were in between to those in rice and the Triticeae species included in the analysis (Table S14; Figures 1d and 2a). The chromosome synteny and derivation among these species showed interesting patterns. All seven chromosomes in orchardgrass corresponded strongly (~80%) to the 12 rice chromosomes (Table S16). Specifically, orchardgrass chromosome (CDgl) 4 and CDgl 7 were syntenic to rice chromosome (COsa) 1 and COsa 5 (Table S17), and two ends of CDgl 4 corresponded to the opposite ends in COsa 1 (Figure S9). In *A. tauschii* chromosomes (CAta), over 50% of CDgl 3, 4, 6 and 7 had syntenic matches to CAta 2, 3, 7 and 1, respectively, indicating that these chromosome pairs were conserved after divergence of orchardgrass and *A. tauschii*. The results further suggested possible chromosome fusions in the species ancestral to orchardgrass or chromosome divergence in the species ancestral to rice.

**Figure 3:**
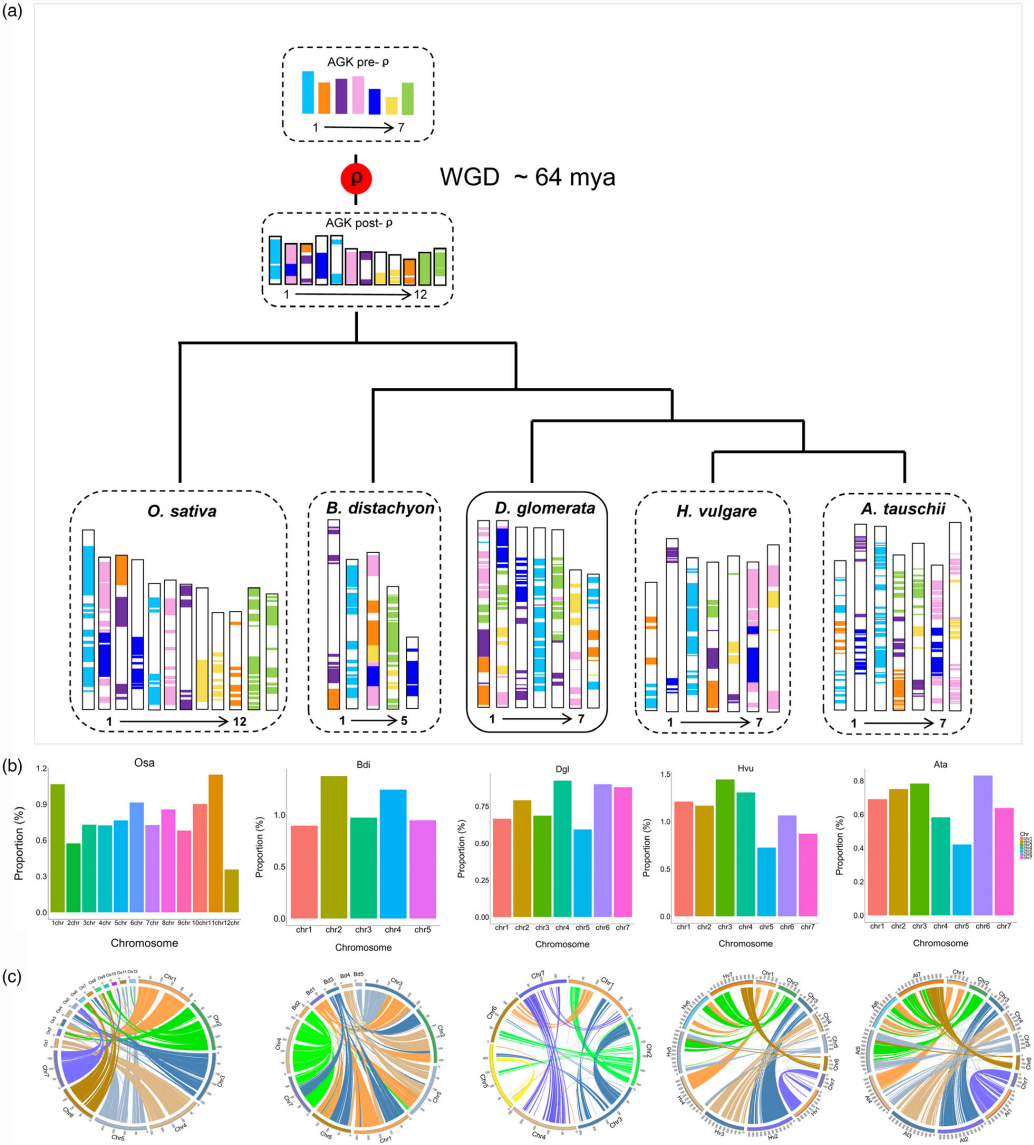
Modern chromosome derivation in orchardgrass and four other grass species. (a) Modern chromosome derivation in orchardgrass, *O. sativa*,* B. distachyon*,* H. vulgare* and *A. tauschii* from ancestral chromosomes. AGKindicates the ancestral grass karyotype. (b) Proportion of monocot‐specific genes (relative to dicot genes) to all genes on each chromosome in the five grass species. The conserved chromosomes chr1 and chr5 in *O. sativa*, chr2 in *B. distachyon*, chr4 in orchardgrass, chr3 in *H. vulgare* and chr3 in *A. tauschii* had higher monocot‐specific gene proportions than other chromosomes. (c) Circos plot of regions of orchardgrass syntenic to *O. sativa*,* B. distachyon*, orchardgrass, *H. vulgare* and *A. tauschii*.

To reveal chromosome rearrangements in orchardgrass, we used the approach describing grass karyotype (AGK) genes by Murat *et al*. (2017). A total of 11 401 orchardgrass AGK genes were identified, accounting for 28.44% of all genes, lower than the percentage in *B. distachyon* (47.47%) and rice (30.05%) and higher than that in *A. tauschii* (23.63%) and *H. vulgare* (16.37%) (Table S18). The AGK gene composition of each CDgl wasmuch more complex than that in the other four species (Figure 3a). In particular, CDgl 4 and 6 contained AGK genes from two ancient chromosomes (AChrs), while the AGK genes in the other four CDgls were from more than two AChrs, suggesting possible extensive transposon accumulations or alterations of chromosomal localization during the speciation of orchardgrass. Specifically, each grass species comprised one evolutionarily conserved chromosome, of which almost all AGK genes came from ancient chromosome 1, such as AGK genes on COsa 1 and 5, *B. distachyon* chromosome (CBdi) 2, CDgl 4, *H. vulgare* chromosome (CHvu) 3 and CAta 3 (Figure 3a). The conserved chromosomes from each grass species had a higher monocot‐specific gene proportion than other chromosomes (Figure 3b; Table S19), indicating that these evolutionarily conserved chromosomes contain genes that are essential for monocot species development and that these genes may have been protected from chromosome disturbance during the speciation of monocots.

To clarify when orchardgrass underwent whole‐genome duplication, synonymous substitutions (ks) were characterized in rice, *B. distachyon* and orchardgrass. The peak ks was 0.5 for orthologous gene pairs between orchardgrass and rice and 0.3 between orchardgrass and *B. distachyon* (Figure 2b), indicating that a whole‐genome duplication event occurred before the divergence of orchardgrass, rice and *B. distachyon*, with one duplication event approximately 64 Mya in orchardgrass (Figure 3c).

### Gene family analysis

In the monophyletic group (orchardgrass, *B. distachyon*,barley, *T. urartu*, rice and *A. tauschii*), 8797 gene families were shared while 1170 gene families were specific to orchardgrass (Figure 2a,c). The gene families unique to orchardgrass were involved in starch, sucrose metabolism, fatty acid metabolism and nitrogen compound metabolic processes. This is not surprising, given the roles of these products in ruminant digestion of forage grass (Chamberlain *et al*., 1993; Daley *et al*., 2010; Tamminga *et al*., 1991). Hormone signal transduction, photosynthesis, plant–pathogen interaction and ABC transport pathway gene families were also specifically detected in orchardgrass, which may contribute to development and resistance to biotic/abiotic stress (Kang *et al*., 2011; Tables S20 and S21).

Orchardgrass shared a common ancestor with three Triticeae species, and the lineages diverged between 17.5 and 27.6 Mya (Figure 2a). Compared to the Triticeae species, orchardgrass contained 128 gene families that substantially expanded and 11 gene families that substantially contracted (Figure 2a). The expanded families were enriched in four pathways: galactose metabolism, starch and sucrose metabolism, sesquiterpenoid and triterpenoid biosynthesis, and brassinosteroid biosynthesis (Tables S22 and S23). The families involved in galactose metabolism and starch and sucrose metabolism were the CELL WALL INVERTASE (CWINV) family (17 genes in orchardgrass versus seven genes in rice), ALDOSE 1‐EPIMERASE (AEP) family (13 versus six) and GALACTINOL SYNTHASE (GOLS) family (10 versus two). The expansion of these families may contribute to the nutritional quality of orchardgrass and its development as a forage (Chamberlain *et al*., 1993; Tamminga *et al*., 1991; Table S24). Triterpenoids are a component of wax that are often related to drought resistance (Seo *et al*., 2011; Zhu and Xiong, 2013). In orchardgrass, there was a substantial expansion in sesquiterpenoid and triterpenoid biosynthesis genes (Table S24), where orchardgrass had more GERMACRENE D SYNTHASE (GDSY) genes than rice (eight vs. two). In addition, some families were enriched in the biosynthesis of brassinosteroids that may regulate lateral tiller formation in perennial forage grasses (Zaman *et al*., 2016). Among them, orchardgrass had more BRASSINOSTEROID INSENSITIVE (BRI) and BRASSINOSTEROID‐SIGNALLING KINASE (BSK) genes than rice (six vs two for BRI and six vs three for BSK; Table S24). Although there are many possibilities, the reasons for these gene expansions in orchardgrass are unclear.

The family members of TFs were compared among orchardgrass and five closely related Poaceae species (Table S25). The number of B3 family members was approximately three‐ to sevenfold higher in orchardgrass (385) than in other species, and most of them (90.39% or 348/385) were from the PRODUCTIVE MERISTEM (REM) family (Table S26). REM genes are related to vernalization, which is critical in perennial cool‐season grasses such as orchardgrass (Mantegazza *et al*., 2014; Moser and Hoveland, 1996; Romanel *et al*., 2009). In orchardgrass, most REM genes were highly expressed specifically in flowers and spikes compared with other tissues, and all REM genes were expressed dynamically during the flowering process (Figure S10a,b). Additionally, the expansion peak of the REM genes that occurred between 2 and 3Mya overlapped with the Pleistocene epoch beginning 2.58 Mya (Figure S10c), indicating that the ice age conditions during the Pleistocene epoch might have contributed to REM gene expansion to optimize reproduction, allowing orchardgrass to adapt to stressful conditions. A higher density of TE/LTRs was detected in the downstream region of REM genes than in the other genes in orchardgrass, suggesting potential regulation of REM genes by transposons (Figure S10d).

### Population structure and diversity

To understand the genetic diversity and population structure of orchardgrass, we resequenced 76 diploid and autotetraploid accessions collected worldwide (Tables S27–S30). Three main clusters were generated in the phylogenetic tree based on the resequencing data (Figure S11). The three clusters containing wild accessions corresponded to three geographical regions: Western Mediterranean (Cluster 1), Eastern Mediterranean/Central Asia (Cluster 2) and East Asia/Northern Europe (Cluster 3). As accessions from East Asia/Northern Europe were grouped into one cluster, they may have intercrossed historically despite a large geographical separation, possibly through trade routes between Asia and Europe, such as the Silk Road (Li *et al*., 2015). The group containing both wild and cultivated orchardgrass populations had a complex subpopulation structure (Figure S12), which was likely a result of the outcrossing nature of orchardgrass (Xie *et al*., 2014). To eliminate biases in single nucleotide polymorphism (SNP) calling caused by mixed polyploids, only 43 autotetraploid genotypes were selected to accurately characterize the structure and diversity of the cultivars and wild materials. The autotetraploid cultivars and wild genotypes were not separated *via* principal component analysis (PCA) and phylogenetic analyses, and their genetic diversities were similar (Figures S13 and S14; Table S31), suggesting a short history of domestication and that domestication did not have a strong impact on the genetic diversity of orchardgrass (Casler *et al*., 2001; Xie *et al*., 2014).

### The genomic basis of flowering regulation

**Figure 4:**
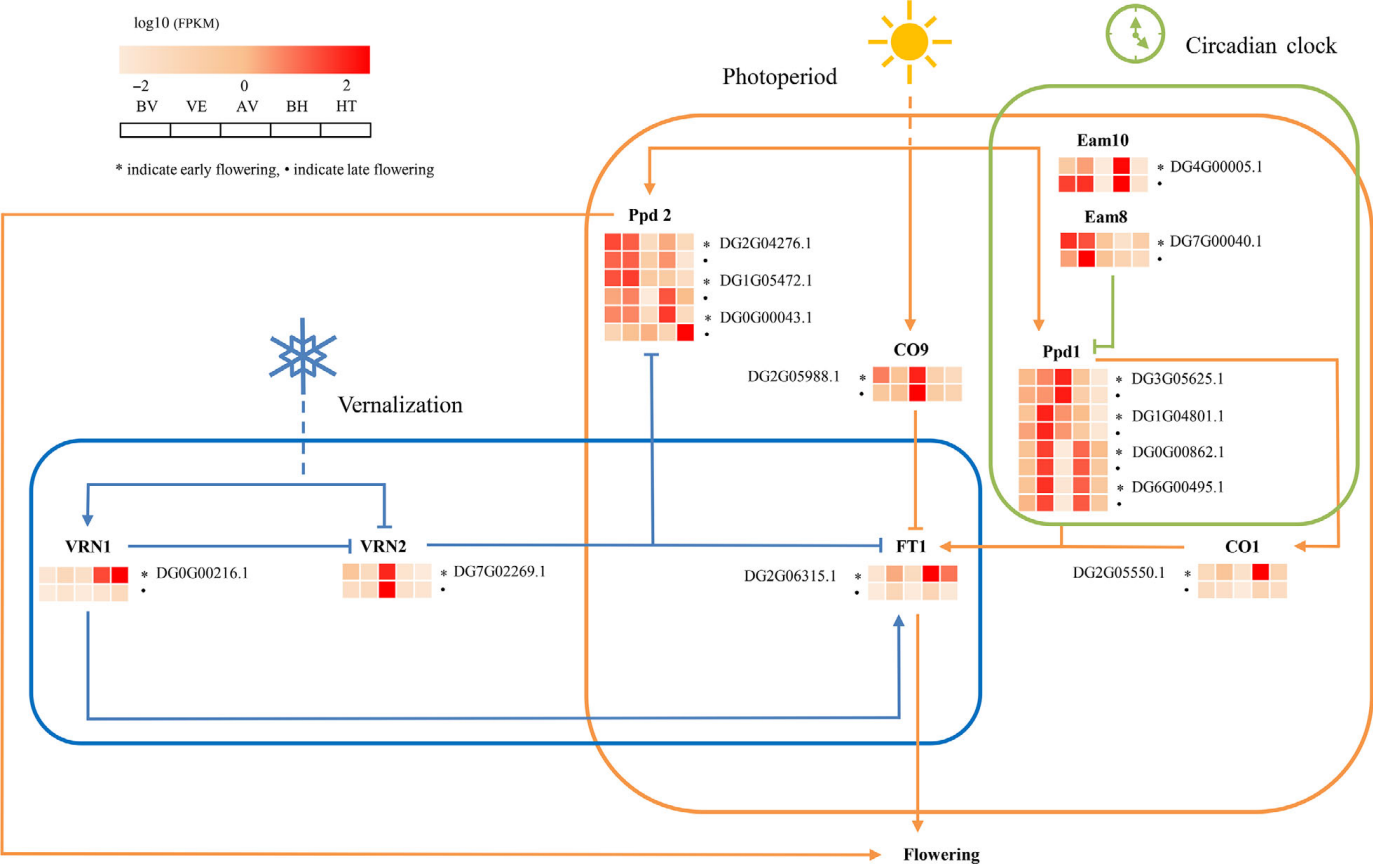
A simplified representation of the flowering pathway in *D. glomerata*. The blue, orange and red lines indicate genes related to the vernalization pathway, photoperiod pathway and circadian clock pathway, respectively. Arrows indicate positive regulation, and lines with bars indicate negative regulation. The heatmap shows the relative expression of candidate genes in different stages. Early and late phenotypes are indicated by asterisks and dots, respectively.

Floweringtime is a critical trait related to environmental adaptation in higher plants (Simpson and Dean, 2002; Zhang *et al*., 2009). Heading date is a surrogate measure of flowering time and is strongly correlated with the yield and quality of forage grasses (Sheldrick *et al*., 1986; Bushman *et al*., 2012). In this study, 603 orthologues and paralogues in the orchardgrass genome were identified, corresponding to 210 flowering‐related genes in the *Arabidopsis thaliana* flowering‐time gene data set (Table S32; Bouché *et al*., 2016). Of these, 85 orchardgrass orthologues and paralogues corresponding to 53 flowering‐related genes were differentially expressed between early‐ and late‐flowering lines, and 25 and five were detected in the vernalization and photoperiod pathways, respectively (Table S33). Several key flowering regulators such as the photoperiod gene *CO1*, vernalization genes *VRN1* and *VRN2*, circadian clock gene *LUX1* and flowering integrator *FT* paralogue were differentially expressed between early‐ and late‐flowering lines, potentially contributing to the difference in heading date (Figure S15a). Additionally, five FT orthologues might have undergone expansion during orchardgrass evolution, suggesting their essential roles in floweringtime (Figure S15b). Based on these findings, we constructed a simplified flowering pathway in orchardgrass (Figure 4; Drosse *et al*., 2014).

**Figure 5:**
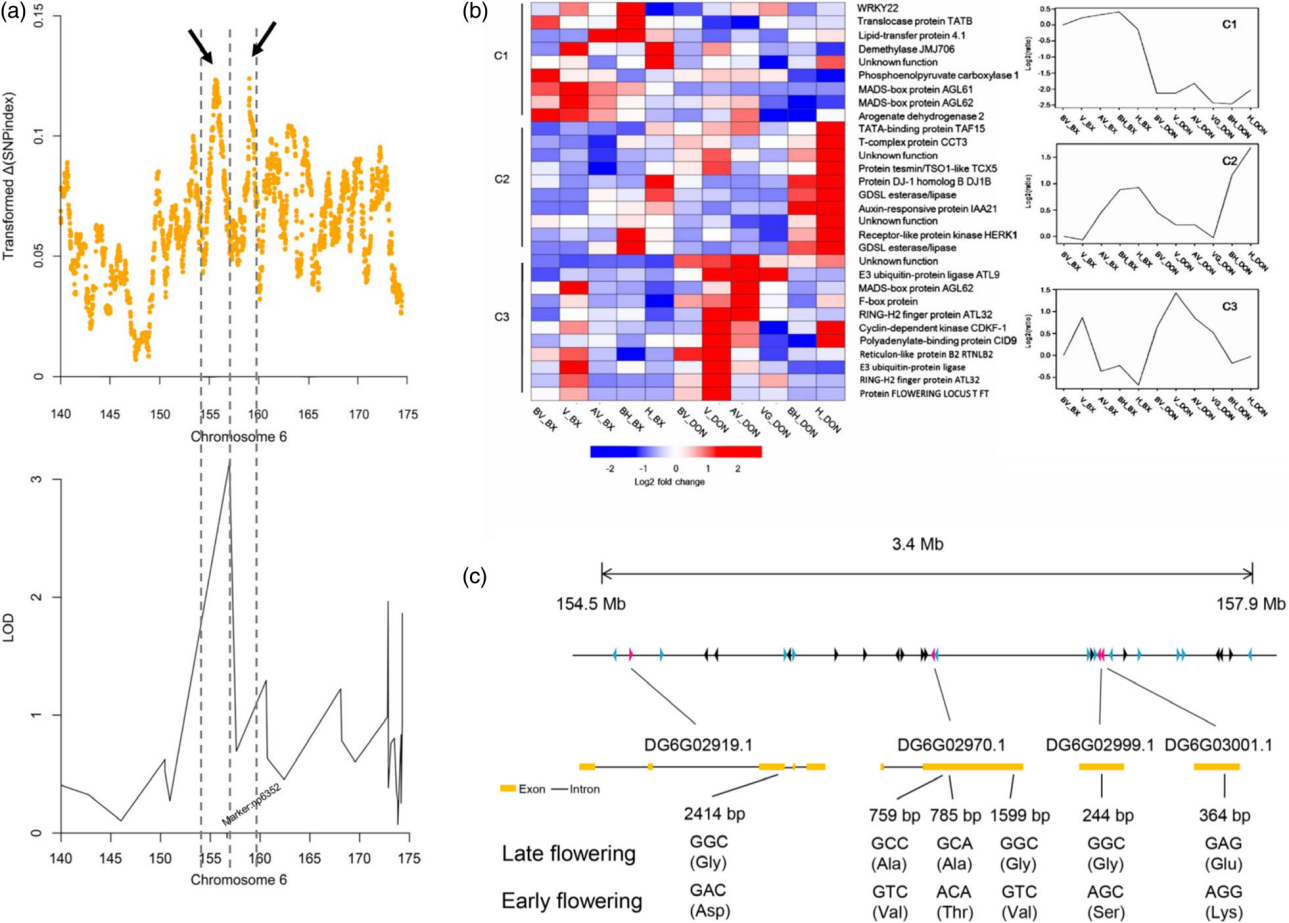
Genetic mapping of the orchardgrass flowering‐related candidate genes. (a) Mapping the flowering‐time genes by BSA and QTL analysis. The transformed ∆(SNP index) is the product of the ∆(SNP index) and normalized SNP density in each 1‐Mb sliding window (10‐kb steps).The dark arrow and dashed line indicate the positions of the 1.89‐ and 2.55‐Mb peaks, respectively. (b) The clusters and expression patterns of 30 candidate genes. The heatmap on the left side shows the expression of 30 candidate genes, and the line chart on the right side shows the expression pattern of clusters. (c) Exon–intron structure and nonsynonymous SNPs of four candidates in two phenotypes.

To identify candidate genetic regions and key regulators associated with heading date, we integrated QTL analysis and BSA with transcriptome expression‐profiling data. The peak value for the transformed ∆(SNP index) localized to two regions spanning from 154.344 to 156.231 Mb and from 157.05 to 159.599 Mb on chromosome 6. Based on the QTL results, we also identified a major locus at 157.639 Mb (np6325) on chromosome 6 that overlapped with the BSA candidate regions (Figure 5a). Fine‐mapping analysis identified a 4.426‐Mb overlapped region on chromosome 6 that may harbour the major locus contributing to orchardgrass heading date. We scanned for nucleotide diversity, differentiation and linkage disequilibrium (LD) to determine whether this region was under selection. No significant difference in nucleotide diversity(π), F_ST_or LD was observed between wild and cultivated accessions, implying that this candidate region was not under selection (Figure S16). The artificial domestication history of orchardgrass is relatively short in comparison with that of other forages, and extensive variation in flowering time may be attributed to adaptation to complex environments. After removing genes that were not expressed among the prevernalization, vernalization, postvernalization, preheading and heading stages, 30 candidate genes were predicted within this region (Figure 5b, Table S34). Polymorphism detection identified 6 nonsynonymous SNPs corresponding to 4 candidates, including one *FT‐like* gene and three MADS‐box genes, in the early‐ and late‐flowering populations (Figure 5c). In previous reports, the MADS‐box family was revealed to be a highly conserved gene family involved in flowering time, floral organ formation and inflorescence architecture (Gramzow and Theißen, 2015; SchilLing *et al*., 2018). In the orchardgrass reference sequence, we identified 94 MADS‐box genes, including 58 type I and 36 type II genes (Gramzow and Theissen, 2010; Table S35). The MADS‐box gene family was markedly expanded in the orchardgrass genome (Table S35) compared with other grass genomes, which likely drives the extensive variation in heading date and strong adaptability to environmental conditions of orchardgrass.

To investigate the gene expression of these four candidates, comparative transcriptome analysis was performed between the early‐flowering and late‐flowering orchardgrass lines. Gene model DG6G02970.1 was the only significantly differentially expressed gene; this gene encodes the MADS‐box gene *AGL61*‐like, which plays an essential role in pollen tube guidance and the initiation of endosperm development (Steffen *et al*., 2008). Mutants of the *A. thaliana* homologue AT2G24840.1 (*AGAMOUS‐LIKE 61, AGL61*) have a phenotype associated with female fertility reduction and defective central cells with abnormal morphology. *AGL61‐like* showed higher expression among five critical flowering stages in the early‐flowering line than in the late‐flowering line (Figure S17). Three nonsynonymous SNPs were identified in the *AGL61‐like* gene, resulting in changes from alanine to valine, alanine to threonine and glycine to valine (Figure 4c). Thus, DG6G02970.1 might participate in flowering regulation of orchardgrass.

Weighted gene co‐expression network analysis (WGCNA) was used to search for candidate genes that were associated with flowering regulators. A total of 8629 differentially expressed genes (DEGs) between early‐ and late‐flowering lines were chosen as probes for WGCN construction, of which genes in three modules (pink, purple and green modules) were related to the vernalization response (Figure S18, Table S36), including 5 *CONSTANS*‐LIKE and 3 *FT*‐LIKE genes. In cereal crops, *VRN2* is a flowering repressor that is down‐regulated by VRN1 (Andrew and Jorge, 2012). *VRN2* is associated with a set of 176 genes in orchardgrass (magenta module) (Table S37). In this module, several known flowering genes were detected, including *ARR9/3/1*,* CONSTANS*/*CONSTANS*‐LIKE, *LHY* and *PRR37*, which are involved in the circadian clock and photoperiod signalling pathways (Suárezlópez *et al*., 2001). The gibberellic acid (GA) and abscisic acid (ABA) pathway‐related genes *GA20ox1D*,* GA20ox2*,* PYL5* and *ABI5*were also identified, which have been shown to play critical functions in flowering modulation in *A. thaliana* (Andrew *et al*., 2012; Kim *et al*., 2014; Wang *et al*., 2013).

Remarkably, when analysing the gene expression in early‐ and late‐flowering lines, many genes in this magenta module showed different expression profiles at the postvernalization stage (Figure S19). We further identified 38 DEGs between early‐ and late‐flowering lines (Table S38), including genes involved in photosynthesis, chlorophyll catabolic process, sodium ion transport and hormone signal transduction. WGCNA revealed that DG6G02970.1 (*AGL61‐like*) is associated with a set of 114 genes in the early‐flowering line (Table S39). Gene Ontology (GO) term enrichment indicated that carbohydrate metabolic process genes were particularly enriched, and glycolysis/gluconeogenesis pathway genes were enriched in the Kyoto Encyclopedia of Genes and Genomes (KEGG) analysis. Among the biological processes, four terms related to carbohydrate metabolic process and two terms related to response to oxidative stress were highly enriched. The need for a high level of carbohydrates for enhanced flowering has been demonstrated. Carbohydrate accumulation is related to the transition from vegetative growth to flowering (Kozłowska *et al*., 2007). Assuming a conserved function of *AGL61‐like* in flowering regulation, we annotated genes that were differentially expressed inprevernalization stage versus postvernalization stage or preheading stage versus heading stage comparisons in the early‐flowering line. This analysis identified a potential relationship between *AGL61‐like* and the carbohydrate metabolic process. However, transgenic evidence needs to be provided to further confirm that the difference in heading date is caused by *AGL61‐like* alone or the cooperation of *AGL61‐like* and other co‐expressed genes.

## Discussion

Forage grasses are very important for feeding livestock. However, genetic research on these grasses is currently hampered by the lack of a reference genome, which is due to the very large size, high heterozygosity and repetitive sequences of the genomes of these species (Hegde *et al*., 2000; Kawube *et al*., 2015). Here, we assembled a high‐quality reference genome sequence for orchardgrass with a contig N50 value of 0.93 Mb, a scaffold N50 of 6.08 Mb and a super‐scaffold N50 of 252.52 Mb, which covered 94.85% of the estimated genome size. The quality of this reference genome was much higher than that of the latest published forage grass genome for perennial ryegrass in terms of the contig N50 (1637 kb) and scaffold N50 (7006 kb; Byrne *et al*., 2016) and is better than some recently sequenced genomes of crops such as pearl millet (*Pennisetum glaucum* L. Varshney *et al*., 2017), barley (Mascher *et al*., 2017) and *T. urartu* (Ling *et al*., 2018). The high quality of our assembly can be attributed to the use of the unique combination of PacBio SMRT sequencing (Roberts *et al*., 2013), new library construction with the 10× Genomics method (Goodwin *et al*., 2016), and BioNano (Staňková *et al*., 2016) with chromosome‐scale scaffolding via Hi‐C (Belton *et al*., 2012). The latter two technologies were key to resolving the linear order of scaffolds on the chromosomes (Belton *et al*., 2012; Staňková *et al*., 2016; Zhang *et al*., 2018). The orchardgrass genome sequence provides an important resource for future molecular breeding and evolutionary studies.

Forage grass is a principal group of Poaceae grasses (Gibson, 2009), but the performance of forage grass in the evolutionary history of Poaceae is still obscure. In this study, orchardgrass was found to have diverged after rice and before three Triticeae species (*H. vulgare*,* T. urartu* and *A. tauschii*) that seem to have common ancestors with orchardgrass. This phylogenetic relation potentially corresponds to the genome size and LTR expansion time of orchardgrass, which were intermediate to those of rice and the three Triticeae species (Table S14; Figures 1d and 2a). Evolutionarily conserved chromosomes were also detected by analysing ancient chromosome rearrangements in these grass species, such as AGK genes on CDgl 4 corresponding to COsa 1, COsa 5, CHvu 3 and CAta 3 (Figure 3a). Thus, orchardgrass genome information will help clarify the evolutionary processes in Poaceae species, and it provides primary knowledge of the evolutionary status of forage grass among major crops. Orchardgrass has a widespread distribution and good adaptation to many natural environments, which can provide important abiotic/biotic stress resistance genetic resources, aiding in the genetic improvement of rice and Triticeae species.

In all of the plants investigated, TEs comprised the vast majority of all DNA. The activation of TEs frequently causes their duplication and insertion, leading to an increase in genome size (Levin and Moran, 2011). Most contributions to genome size were made by a class of mobile DNA sequences called retroelements, primarily LTR retrotransposons (LTR‐RTs; SanMiguel *et al*., 1996; Vicient *et al*., 1999). Waves of expansion and contraction in numbers of TEs can induce deletions, inversions, translocations and other rearrangements in chromosomes (Yu *et al*., 2011). In addition to these gross effects on the overall architecture of genomes, genome restructuration mediated by TE activity is also essential for the stress response of hosts, facilitating the adaptation of species to changing environments (McClintock, 1983). Evidence from rice suggests that the overall number of stress‐induced genes can be increased by TE activity to help rice adapt to stress (Lisch, 2013). In the present study, LTR‐RTs accounted for 59.42% of the orchardgrass genome (Table S13; Figure 1a). The insertion number of LTR‐RTs reached a peak between 0 and 1 Mya in the Pleistocene (or ice) age, lasting from 2.58 Mya until 10 000 years ago. During the Pleistocene epoch, the large grasslands and savannas of North America expanded and contracted many times. However, during periods of maximum glacial extent, the freezing weather and limited global atmospheric CO_2_ (180 ppm) seriously affected the growth and development of grasslands as well as trees, shrubs and other types of vegetation (Cerling, 1999). To survive during this cold period, plants had to adjust to the novel conditions through molecular or phenotypic plasticity (Nicotra *et al*., 2010). Therefore, the expansion of LTR‐RTs in orchardgrass might be a strategy to confront extreme environmental conditions.

Flowering is a key event in the plant life cycle. Variation in flowering time is a salient feature in the evolution, adaptation and domestication of the grass family (Poaceae).The high‐quality orchardgrass reference genome helps identify flowering‐related homologous genes and additional candidates underlying flowering regulation. This orchardgrass genome and its companion resources will provide resources for Poaceae evolution and diversity studies and allow diploid orchardgrass to serve as a model for studying other forage grass species. The reference genome and large set of SNP markers will accelerate marker‐facilitated trait mapping through genomewide association studies and genomic selection of orchardgrass. The orchardgrass genome sequence and online database will support crop improvement efforts and help identify additional candidate genes underlying biotic and abiotic stress resistance and regulatory pathways controlling growth, flowering, seed production and regeneration in tissue culture—all of which are important traits for sustained agricultural production and meeting the demands for human consumption.

## Experimental procedures

### Sample collection for genome sequencing

The diploid orchardgrass accession 2006‐1 (2n* *= 14) was used for genome sequencing. Accession 2006‐1 was originally collected from Wuxi, Chongqing, China (altitude: 2475m, 31°35.086′N, 109°0.84′E), and is maintained at Sichuan Agriculture University (30°42′N, 103°51′E; Wenjiang, Chengdu; annual mean temperature: 16.0 °C, and annual mean precipitation: 865.9 mm).

### DNA extraction and library preparation

Genomic DNA was extracted from young 2006‐1 leaves using a DNAsecure Plant Kit (TIANGEN, Beijing, China). For PacBio Sequel sequencing, a 20‐kb‐insert‐size SMRTbell library was prepared following the manufacturer's protocol (PacBio, CA). For Illumina (San Diego, CA) short‐read sequencing, libraries were size‐selected for PE150 sequencing. Sequencing libraries with insert sizes ranging from 250 to 350 bp were constructed and sequenced using an Illumina HiSeq X Ten platform at the Novogene Bioinformatics Institute, Beijing.

The GEM reaction and library preparation for 10X Genomics sequencing were conducted using 1ng of input DNA that was size‐selected to have an approximately 50‐kb length. Libraries were barcoded and paired‐end‐sequenced with the Rapid method on an Illumina HiSeq X Ten platform.

### Genome assembly

We constructed a *de novo* assembly of the 2006‐1 genome by combining sequences from four different technologies: Illumina PE150 short‐read sequencing, PacBio Sequel long‐read sequencing, 10× Genomics contig spanning and Hi‐C conformational alignment (Figure S1).

*De novo* assembly of the long reads from SMRT sequencing was first performed using FALCON (v3.0) (https://github.com/PacificBiosciences/FALCON/) and FALCON‐Unzip (Chin *et al*., 2016). Initially, the 55 subreads with the greatest coverage were selected as seed reads to correct for error. The error‐corrected reads were aligned to each other and assembled into genomic contigs using FALCON, with the length_cutoff_pr = 5000, max_diff = 120 and max_cov = 130 parameters. After the initial assembly, FALCON‐Unzip was used to produce primary contigs (p‐contigs), which were polished using Quiver (Chin *et al*., 2013). Subsequently, BWA‐MEM was implemented to align the 10× Genomics data to the assembly using the default settings (Li, 2014). Scaffolding was performed by FragScaff with the barcoded sequencing reads (Adey *et al*., 2014; Appendix S1).

For construction of a BioNano genome map, healthy young leaves of *D. glomerata* were prepared, and high molecular weight DNA isolation, sequence‐specific labelling of megabases of gDNA by nicking, labelling, repairing and staining (NLRS), and chip analysis were performed according to the manufacturer's instructions (BioNano Genomics). The enzyme Nt.BspQI with an appropriate label density (14.5 labels per 100 kb) was selected and applied to digest long‐range DNA fragments. After filtering the molecules with a cut‐off at a minimum length of 150kb, 212Gb of BioNano mapping molecules with an average length of 305.39kb was collected. Then, the RefAligner and Assembler programs in Solve tools (https://bionanogenomics.com/support/software-downloads?_sft_download-type=saphyr) were used to assemble these BioNano molecules, resulting in consensus maps with a total length of 2.58 Gb and an N50 length of 1.55 Mb. These consensus maps were then used to join the assembled scaffolds to form super‐scaffolds.

Two Hi‐C libraries were prepared as described previously (Lieberman‐Aiden and Dekker, 2009). The *de novo* PacBio assembly and Hi‐C library reads were used as input data for further assembly using HiRise, a pipeline designed specifically for assembling the scaffold genome using proximity ligation data (Putnam *et al*., 2016). Hi‐C library sequences were aligned to the draft input assembly using a modified SNAP read mapper (http://snap.cs.berkeley.edu; Zaharia *et al*., 2011). The separations of Hi‐C read pairs that mapped within draft scaffolds were analysed by HiRise to generate a likelihood model for genomic distance between read pairs, and the model was used to identify and break putative mis‐joins, to score prospective joins and to select joins above a threshold (Appendix S1).

To evaluate the quality of the V1 assembly, we compared the V1 assembly to BioNano super‐scaffolds using NUCmer in the MUMmer package (Delcher *et al*., 2002). Then, we drew a dot plot using mummerplot in the same package with default parameters.

### Annotation of repetitive sequences

TEs in the orchardgrass genome were annotated by combining *de novo*‐based and homology‐based approaches. For the *de novo*‐based approach, we used RepeatModeler (http://www.repeatmasker.org/RepeatModeler.html), LTR_FINDER (http://tlife.fudan.edu.cn/ltr_finder/) and RepeatScout (http://www.repeatmasker.org/) to build the *de novo* repeat library. For the homology‐based approach, we used RepeatMasker (http://www.repeatmasker.org, version 3.3.0) against the Repbase TE library and RepeatProteinMask (http://www.repeatmasker.org/) against the TE protein database (Chen, 2009; Price *et al*., 2005; Xu and Wang, 2007; Appendix S1). Spearman correlation analyses were conducted to test for correlations between genome size and the proportion of TEs in the following ten species: rice, *T. urartu*,* B. distachyon*, barley, *A. tauschii*,* Setaria italica*,* Sorghum bicolor*,* Zea mays*,* D. glomerata* and *A. thaliana*.

For the intact LTR‐RTs, we aligned thesequences between the 5′ and 3′ LTRs using MUSCLE (Edgar, 2004). Nucleotide variations (λ) in the 5′ and 3′ ends of intact LTR‐RTs were calculated, and DNA substitution rates (*K*) were calculated by *K* = −0.75ln(1–4λ/3). The insert time of LTR‐RTs was estimatedusing the formula *T *= *K*/2*r* (*r* = 1.3 × 10^−8^ per site and per year; Ma and Bennetzen, 2004).

### Gene prediction

A high‐throughput RNA‐seq analysis was conducted using Illumina short reads from five tissues: root, leaf, stem, flower and spike. In addition, a single library was constructed from a pooled DNA sample of the five different tissues for full‐length transcriptome sequencing using the PacBio Sequel platform. Genes were ascribed through a combination of homologue‐, *de novo‐* and transcriptome‐based predictions. Homologous proteins from four plant genomes (*A. thaliana*, rice, *T. aestivum* and *Z. mays*) were downloaded and aligned to the orchardgrass genome using tblastN (Altschul *et al*., 1990), with an E‐value cut‐off of 1e‐5. The BLAST hits were conjoined by Solar (Yu *et al*., 2006). GeneWise (https://www.ebi.ac.uk/Tools/psa/genewise) was used to predict the exact gene structure of the corresponding genomic regions for each BLAST hit (Homo‐set) (Cook *et al*., 2018). For transcriptome‐based predictions, RNA‐seq data from Illumina were mapped to the assembly using TopHat (http://ccb.jhu.edu/software/tophat/index.shtml, version 2.0.8), followed by Cufflinks (http://cole-trapnell-lab.github.io/cufflinks/, version 2.1.1; Kim *et al*., 2013). In addition, PacBio RNA‐seq data were used to create pseudo‐ESTs, which were also mapped to the assembly. Gene models were predicted by PASA (http://pasapipeline.github.io/). This gene set was denoted the PASA‐T‐set and was used to train *ab initio* gene prediction programs. The *ab initio* gene prediction programs Augustus (http://augustus.gobics.de/, version 2.5.5), GENSCAN (http://genes.mit.edu/GENSCAN.html, version 1.0), GlimmerHMM (http://ccb.jhu.edu/software/glimmerhmm/, version 3.0.1), geneid (http://genome.crg.es/software/geneid/) and SNAP (http://korflab.ucdavis.edu/software.html) were used to predict coding regions in the repeat‐masked genome (Blanco *et al*., 2007; Burge and Karlin, 1998; Keller *et al*., 2011; Majoros *et al*., 2004). Gene model evidence from Homo‐set, Cufflinks‐set, PASA‐T‐set and the *ab initio* programs was combined using EVidenceModeler (EVM) (http://evidencemodeler.sourceforge.net/) into a nonredundant set of gene structures (Haas *et al*., 2008). Functional annotation of protein‐coding genes was achieved using BLASTP (E‐value 1e‐05) against two integrated protein sequence databases (Altschul *et al*., 1997): Swiss‐Prot (http://web.expasy.org/docs/swiss-prot_guideline.html) and NR (https://www.ncbi.nlm.nih.gov/). Protein domains were annotated by searching against the InterPro ((http://www.ebi.ac.uk/interpro/, V32.0) and Pfam (http://pfam.xfam.org/, V27.0) databases, using InterProScan (V4.8) and HMMER (http://www.hmmer.org/, V3.1), respectively (Finn *et al*., 2017, 2015, 2013; Zdobnov and Apweiler, 2001). The GO (http://www.geneontology.org/page/go-database) terms for each gene were obtained from the corresponding InterPro or Pfam entry. The pathways that the genes may be involved in were determined through a BLAST search against the KEGG database (http://www.kegg.jp/kegg/kegg1.html, release 53) with an E‐value cut‐off of 1e‐05 (Appendix S1). It was recently shown that Repbase contains some R‐gene domains and using it for masking may result in under‐annotation of R genes (Bayer *et al*., 2018), and BLASTP was performed between homologous protein‐coding genes and TE‐filter protein‐coding genes. If the coverage of homologous species protein sequences was >0.5 and the coverage of TE‐filter protein sequences was >0.7, these TE‐filter protein sequences would be added to the final protein‐coding genes.

### Constructing gene families

The protein sequences from *A. thaliana*,* Populus trichocarpa*, rice, *S. bicolor*,* Z. mays*,* S. italica*,* B. distachyon*,* H. vulgare*,* T. urartu*,* A. tauschii*,* Elaeis guineensis* and *Musa acuminata* were downloaded from Phytozome 12 (https://phytozome.jgi.doe.gov/pz/portal.html) and the NCBI (https://www.ncbi.nlm.nih.gov/). Across the species that were included, when multiple transcripts were present in one gene, only the longest transcript in the coding region was included in further analysis. Additionally, genes encoding proteins with fewer than 50 amino acids were removed. The filtered blast results were obtained between all species’ protein sequences through BLASTP with an *E*‐value of 1e‐5. Protein sequences from all 13 species were clustered into paralogous and orthologous groups using OrthoMCL (http://orthomcl.org/orthomcl/) with an inflation parameter equal to 1.5.

### Phylogenetic tree reconstruction

Protein sequences from single‐copy gene families were aligned using MUSCLE (Edgar, 2004), and the alignments of each gene family were concatenated to a super‐alignment matrix. A phylogenetic tree was constructed using RAxML (http://sco.h-its.org/exelixis/web/software/raxml/index.html) with the maximum‐likelihood method and a bootstrap value of 100, where *A. thaliana* and *P. trichocarpa* were designated as outgroups. A Venn diagram was constructed to display the number of gene families that were shared among six Poaceae species (orchardgrass, *B. distachyon*,* H. vulgare*,* T. urartu*, rice and *A. tauschii*) clustered into one group of the phylogenetic tree.

### Species divergence time estimation

The MCMCTree program (http://abacus.gene.ucl.ac.uk/software/paml.html) was implemented in Phylogenetic Analysis with Maximum Likelihood (PAML) to infer the divergence time of the nodes on the phylogenetic tree. The MCMCTree parameters were as follows: a burn‐in of 10 000 steps, sample number of 100 000 and sample frequency of 2. The following calibration times of divergence were obtained from the TimeTree database (http://www.timetree.org/): 120.0–155.8 Mya for *A. thaliana* and rice, 105.0–124.7 Mya for riceand *M. acuminata*, 39.4–53.8 Mya for riceand *B. distachyon*, 3.2–5.3 Mya for *T. urartu* and *A. tauschii*, 99.9–118.8 Mya for *A. thaliana* and *P. trichocarpa*, and 22.7–28.5 Mya for *S. italica* and *S. bicolor*.

### Expansion and contraction of gene families

The expansion and contraction of gene families were determined by comparing the cluster size differences between the ancestor and each species using the CAFÉ (v3.1) program (Han *et al*., 2013). A random birth‐and‐death model was used to evaluate changes in gene families along each lineage of the phylogenetic tree. A probabilistic graphical model (PGM) was used to calculate the probability of transitions in each gene family from parent to child nodes in the phylogeny. Using conditional likelihoods as the test statistics, we calculated the corresponding *P*‐values of each lineage, and a *P*‐value of or below 0.05 was considered significant.

To investigate the genes involved in the galactose metabolism, starch and sucrose metabolism, sesquiterpenoid and triterpenoid biosynthesis, and brassinosteroid biosynthesis pathways, genes involved in these processes in *A. thaliana* and *B. distachyon* were downloaded from the NCBI (https://www.ncbi.nlm.nih.gov/; Cao, 2015; Clouse, 2008; Gross and Pharr, 1982; Zheng *et al*., 2014).Using these homologues as queries, the candidate genes in *D. glomerata* were identified by BLASTP with an *E*‐value cut‐off of 1e‐5. The aligned hits with at least 50% coverage of the seed protein sequences and >50% protein sequence identity were designated homologues. Protein domains of these homologues were predicted by Pfam (http://pfam.xfam.org/). Only the genes with the same protein domain were considered homologues.

### Genome synteny and whole‐genome duplication

A homologue search within the orchardgrass genome was performed using BLASTP (*E*‐value < 1e−5), and MCScanX was used to identify syntenic blocks within the genome. For each gene pair in a syntenic block, ks values were calculated, and values of all gene pairs were plotted to identify putative whole‐genome duplication events within *D. glomerata*. The molecular clock rate (*r*) was calculated to be 6.96 × 10^−9^ substitutions per synonymous site per year. The duplication time was estimated using the formula ks/2r (Moniz de Sa and Drouin, 1996). The syntenic blocks between chromosomes were visualized using Circos (Krzywinski *et al*., 2009).

### SNP calling

To identify SNPs found in different orchardgrass accessions, 76 accessions were used to generate high‐quality paired‐end reads, and the reads were mapped to the orchardgrass reference genome using the Burrows‐Wheeler Aligner (BWA) (Li and Durbin, 2009). The alignment results were converted to BAM files using SAMtools (Li and Durbin, 2009). The SNPs were called at a population scale using a Bayesian approach, as implemented in the package SAMtools, and only high‐quality SNPs (coverage depth ≥6, root mean square (RMS) mapping quality ≥20, minor allele frequency (maf) ≥ 0.01 and misses ≤0.2) were kept for subsequent analyses.

To eliminate biases in SNP calling caused by mixed polyploids, SNPs were called for the 43 autotetraploid genotypes at the population level by using GATK (Mckenna *et al*., 2010), and only high‐quality SNPs (coverage depth ≥15, RMS mapping quality ≥20, maf ≥ 0.05 and misses = 0) were kept for subsequent analyses.

### Phylogenetic tree and population structure

A method based on the diploid model was used to build a phylogenetic tree for wild and cultivated genotypes with a mixture of diploid and autotetraploid individuals, a method that has been successfully applied in other polyploid plants (Hirsch *et al*., 2013; Lu *et al*., 2013). An individual‐based neighbour‐joining (NJ) tree was constructed using TreeBeST v1.9.2 (Vilella *et al*., 2009) with 1000 bootstraps. The population genetic structure was examined via Admixture 1.23 (Alexander *et al*., 2009), and the number of assumed genetic clusters K ranged from 2 to 6, with 10 000 iterations for each run. To clarify the phylogenetic relationships of the 43 autotetraploid genotypes from a genomewide perspective, an individual‐based NJ tree was constructed using TASSEL 5.0 (Bradbury *et al*., 2007). PCA and diversity (PiPerBP) estimation were performed in TASSEL 5.0.

### Identification of genes that regulate flowering time

Genes that regulate flowering time are often conserved across divergent species (Blümel *et al*., 2015). Genes that regulate flowering time in *A. thaliana* were retrieved from a recently developed database, FLOR‐ID20 (FLOR‐ID: an interactive database of flowering‐time gene networks in *A. thaliana*), which includes 295 protein‐coding genes. Using the *A. thaliana* homologues as queries, the putative orthologous candidate genes in orchardgrass were identified by BLASTP with an *E*‐value cut‐off of 1e‐5. If these genes were in common families in OrthoMCL, then their protein domains were predicted by Pfam (http://pfam.xfam.org/). Only genes that had the same protein domain as X were considered orthologous to the *A.thaliana* genes.

### Transcriptome analysis

Clean data were obtained by removing reads containing adapter and poly‐N sequences and low‐quality reads from the raw data. High‐quality reads were then mapped to the draft reference genomes by TopHat2 (Kim *et al*., 2013) with the parameters ‐max‐intron‐length 500 000, ‐read‐gap‐length 10, ‐read‐edit‐dist 15, ‐max‐insertion‐length 5 and ‐max‐deletion‐length 5. The expression level (reads per kilobase of transcript per million mapped reads (RPKM) value) of each protein‐coding gene was calculated by HTSeq (Anders *et al*., 2015) using default parameters. DESeq2 (Anders and Huber, 2010) was used to normalize gene expression (BaseMean) in each sample and to identify DEGs for each group that was compared, using ‘*P*‐adj (adjusted *P*‐value) < 0.05’ as the threshold. All DEGs were mapped to GO terms in the GO database (http://www.geneontology.org/). The significantly enriched GO terms were selected by using a hypergeometric test to develop hierarchical clusters of a sample tree by Euclidean distance using topGO (Young *et al*., 2010). To further clarify the biological functions of DEGs, a pathway‐based analysis was conducted using the KEGG database (http://www.genome.jp/kegg). Pathways with *q*‐values < 0.05 were considered significantly enriched. Log2‐normalized RPKM values were used to generate co‐expression networks using the WGCNA package in R (Langfelder and Horvath, 2008). Gene structure analysis was performed by using the TAPIS pipeline. Mapping of high‐quality PacBio reads and identification of alternative splicing (AS) events were performed by GMAP with default settings (Abdelghany *et al*., 2016; Tables S40–S42).

### Bulked segregant analysis

To identify SNPs of genes involved in flowering time, 29 full‐sib individuals from an F_1_ mapping population of 213 lines were used for QTL sequencing (Zhao *et al*., 2016). SNPs that were homozygous in one parent and heterozygous in the other parent were prioritized and extracted from the ‘vcf’ output files. The homozygous genotype of the parent was used as the reference to calculate the number of reads of this parent's genotype in the individuals in the offspring pools. The ratio of reads harbouring the SNP that was different from the reference sequence was calculated as the SNP index of the base site. Sliding‐window methods were used to present SNP indexes across the whole genome. The SNP index for each window was calculated as the average of all SNP indexes in the selected window of the genome. The window size was set as 1 Mb, and the step size was set as 1 Kb. The difference in the SNP index of the two pools, namely one earlier flowering pool and one later flowering pool, was calculated as the transformed Δ(SNP index).

## Data tax

The orchardgrass genome has been deposited under BioProject accession number PRJNA471014. PacBio and Illumina raw reads, resequencing sequence reads and Hi‐C data have been deposited in the Sequence Read Archive (SRA) under study accession number SRP150286. Flowering RNA‐seq data have been deposited under SRA accession numbers SRR5341102 and SRP131899.

## Author contributions

X.Q.Z., L.H., B.B. and W.J. conceived and designed the project and the strategy; L.H., G.F., H.Y., W.J., Z.Y., L.X. and P.C. contributed to plant sample collection, DNA/RNA preparation, library construction and sequencing; L.H., H.Y., G.F., X.Q.Z. and Z.Z. worked on genome assembly and annotation and comparative and population genomic analyses; G.F., L.H., X.X.Z. and Z.Z. performed transcriptome and genetic analyses and identified candidate genes of flowering time; and L.H., H.Y., G.F., B.B., J.W., A.B., M.L., W.J., G.N., W.X. and X.Q.Z. wrote and revised the manuscript.

## Competing interests

The authors declare no competing interests.

## Supporting information


**Figure S1** The orchardgrass genome landscape.
**Figure S2** Workflow of the orchardgrass genome assembly.
**Figure S3** K‐mer frequency distributions in orchardgrass.
**Figure S4** Scaffold Hi‐C contact map data analysis.
**Figure S5** The chromosome number of diploid orchardgrass (genotype 2006‐1).
**Figure S6** Consistency between the Hi‐C and BioNano results.
**Figure S7** The density of TEs surrounding genes.
**Figure S8** The distribution of divergence time for LTRs/Gypsy and LTRs/Copia.
**Figure S9** Synteny analysis of seven chromosomes from orchardgrass (Dgl) to twelve chromosomes from *O. sativa* (Osa) and seven chromosomes from *A. tauschii* (Ata).
**Figure S10** REM family in orchardgrass.
**Figure S11** Phylogenetic tree of 76 orchardgrass accessions.
**Figure S12** Structure analysis of 76 orchardgrass accessions with different K values.
**Figure S13** PCA plot of the first two components (PC1 and PC2) of 43 autotetraploid orchardgrass accessions.
**Figure S14** Phylogenetic tree of 43 autotetraploid orchardgrass accessions.
**Figure S15** Analysis of important flowering‐related orthologues in orchardgrass.
**Figure S16** Nucleotide diversity (π) estimated in wild (red) and cultivated (green) orchardgrass (a) and the *FST* value (b) and patterns of LD in cultivated (c) and wild (d) orchardgrass in the 4.426‐Mb region of orchardgrass chromosome 06.
**Figure S17** Comparison of *AGL61* expression during the five developmental stages in orchardgrass.
**Figure S18** Module‐sample relationship.
**Figure S19** Expression pattern of genes in green, pink and purple modules.

**Table S1** Estimation of genome size.
**Table S2** Sequencing libraries and statistics of the data used for the genome assembly.
**Table S3** Characteristics of orchardgrass assembly containing 7 chromosomes.
**Table S4** SNP location and annotation of assembled orchardgrass genome.
**Table S5** Evaluation of Benchmarking Universal Single‐Copy Orthologs (BUSCO) and gene space coverage using core eukaryotic gene mapping approach (CEGMA) in orchardgrass genome.
**Table S6** Statistics of paired‐end reads mapping.
**Table S7** Assessment of orchardgrass genome using full‐length EST sequences.
**Table S8** Prediction of protein‐coding genes in orchardgrass.
**Table S9** Summary for annotation of predicted protein‐coding genes in the orchardgrass genome assembly.
**Table S10** The mapping information of transcriptome based on PacBio platform.
**Table S11** Mapping summary of RNA‐seq data to the orchardgrass genes.
**Table S12** Noncoding RNAs in the assembly of orchardgrass.
**Table S13** The classification of transposons in orchardgrass genome.
**Table S14** Plant genome size and proportion of TEs in the genome.
**Table S15** Statistics of subgroups in the Copia/Gypsy superfamily (genome ratio %).
**Table S16** The ratio of every seven chromosomes in orchardgrass (Dgl) genome corresponds to Aegilops tauschii (Ata) and Oryza sativa (Osa) genomes.
**Table S17** The ratio of orchardgrass (Dgl) genome corresponds to ratio of Aegilops tauschii (Ata) and Oryza sativa (Osa) genome.
**Table S18** The number of AGK genes and their proportion to all genes in five grass species.
**Table S19** The number of monocot‐specific genes and their proportion of all genes in five grass species.
**Table S20** GO analysis for the unique gene families in orchardgrass.
**Table S21** KEGG pathway of unique families in orchardgrass.
**Table S22** GO analysis for the expanded gene families in orchardgrass.
**Table S23** KEGG pathway of expanded families in orchardgrass.
**Table S24** Four major KEGG enriched pathways of orchardgrass expanded families.
**Table S25** The number of TF family members in six grass species.
**Table S26** The number of B3 subfamily members in six grass species.
**Table S27** The information of resequencing materials.
**Table S28** Summary of data generated on the 79 genotypes of orchardgrass using whole‐genome resequencing.
**Table S29** Summary of mapping rate and coverage of whole‐genome resequencing data.
**Table S30** SNP location and annotation of resequence genotypes.
**Table S31** The genetic diversity from 43 autotetraploid orchardgrass.
**Table S32** Homologous identification of flowering‐related genes in *Dactylis glomerate*.
**Table S33** Differential expressed flowering‐related genes in *Dactylis glomerate*.
**Table S34** Annotation of 30 candidate genes in genomic region found by QTL and BSA.
**Table S35** Expansion of MADS‐box genes in *Dactylis glomerate*.
**Table S36** Annotation of DEGs in three modules associated with vernalization as showed in supplementary note 5.6.
**Table S37** Annotation of DEGs coordinated with VRN2.
**Table S38** Annotation of DEGs between the early‐ and late‐flowering phenotypes.
**Table S39** Annotation of DEGs coordinated with AGL61.
**Table S40** Identification of alternative splicing of transcriptome based on PacBio platform.
**Table S41** GO analysis for the positive genes in orchardgrass.
**Table S42** KEGG pathways of positive genes in orchardgrass.

**Appendix S1** Supplementary note.
