# Supplementary material for: Genome assembly provides insights into the genome evolution and flowering regulation of orchardgrass
Source: Plant Biotechnol J. 2019 Jul 30;18(2):373–88. doi: 10.1111/pbi.13205 (PMC6953241; doi:10.1111/pbi.13205)
Supplement: Supplementary file 1 — Figure S1 The orchardgrass genome landscape. Figure S2 Workflow of the orchardgrass genome assembly. Figure S3 K‐mer frequency distributions in orchardgrass. Figure S4 Scaffold Hi‐C contact map data analysis. Figure S5 The chromosome number of diploid orchardgrass (genotype 2006‐1). Figure S6 Consistency between the Hi‐C and BioNano results. Figure S7 The density of TEs surrounding genes. Figure S8 The distribution of divergence time for LTRs/Gypsy and LTRs/Copia. Figure S9 Synteny analysis of seven chromosomes from orchardgrass (Dgl) to twelve chromosomes from O. sativa (Osa) and seven chromosomes from A. tauschii (Ata). Figure S10 REM family in orchardgrass. Figure S11 Phylogenetic tree of 76 orchardgrass accessions. Figure S12 Structure analysis of 76 orchardgrass accessions with different K values. Figure S13 PCA plot of the first two components (PC1 and PC2) of 43 autotetraploid orchardgrass accessions. Figure S14 Phylogenetic tree of 43 autotetraploid orchardgrass accessions. Figure S15 Analysis of important flowering‐related orthologues in orchardgrass. Figure S16 Nucleotide diversity (π) estimated in wild (red) and cultivated (green) orchardgrass (a) and the FST value (b) and patterns of LD in cultivated (c) and wild (d) orchardgrass in the 4.426‐Mb region of orchardgrass chromosome 06. Figure S17 Comparison of AGL61 expression during the five developmental stages in orchardgrass. Figure S18 Module‐sample relationship. Figure S19 Expression pattern of genes in green, pink and purple modules. [file PBI-18-373-s002.pdf]

Title: Genome assembly provides insights into the genome evolution and flowering regulation of orchardgrass

Linkai Huang[1+], Guangyan Feng[1+], Haidong Yan[1,5+], Zhongren Zhang[2]+, B. Shaun Bushman[3], Jianping Wang[4], Aureliano Bombarely[5], Mingzhou Li[6], Zhongfu Yang[1], Gang Nie[1], Wengang Xie[7], Lei Xu[1], Peilin Chen[1], Xinxin Zhao[1], Wenkai Jiang[2]*, Xinquan Zhang[1]*,

1 Department of Grassland Science, Animal Science and Technology College, Sichuan Agricultural University, Chengdu, 611130, China.

2 Novogene Bioinformatics Institute, Beijing, 100083, China

3 Forage and Range Research Laboratory, USDA-ARS, Logan, UT, 84322, USA

4 Agronomy Department, University of Florida, Gainesville, FL, USA

5 School of Plant and Environmental Sciences, Virginia Tech, Blacksburg, VA 24061, USA.

6 Animal Science and Technology College, Sichuan Agricultural University, Chengdu, 611130, China.

7 State Key Laboratory of Grassland Agro-ecosystems, College of Pastoral Agriculture Science and Technology, Lanzhou University, Lanzhou, 73000, China

+these authors contributed equally to this work

* Corresponding author:

Xinquan Zhang: zhangxq@sicau.edu.cn

Wenkai Jiang: jiangwenkai@novogene.com

Linkai Huang: huanglinkai@sicau.edu.cn

Guangyan Feng: fg62586336@163.com

Haidong Yan: yanhd@vt.edu

Zhongren Zhang: zhangzhongren08@126.com

B. ShaunBushman: Shaun.Bushman@ARS.USDA.GOV

Jiangping Wang: wangjp@ufl.edu

Aureliano Bombarely: aurebg@vt.edu

Mingzhou Li: mingzhou.li@sicau.edu.cn

Zhongfu Yang: yangzf211@163.com

Gang Nie: nieganggrass@hotmail.com

Wengang Xie: xiewg@lzu.edu.cn

Lei Xu: 15928925485@163.com

Peilin Chen: chenpeilin1994@outlook.com

Xinxin Zhao: 824476367@qq.com

Running title: Orchardgrass genome

Supplemental figures include 19 figures

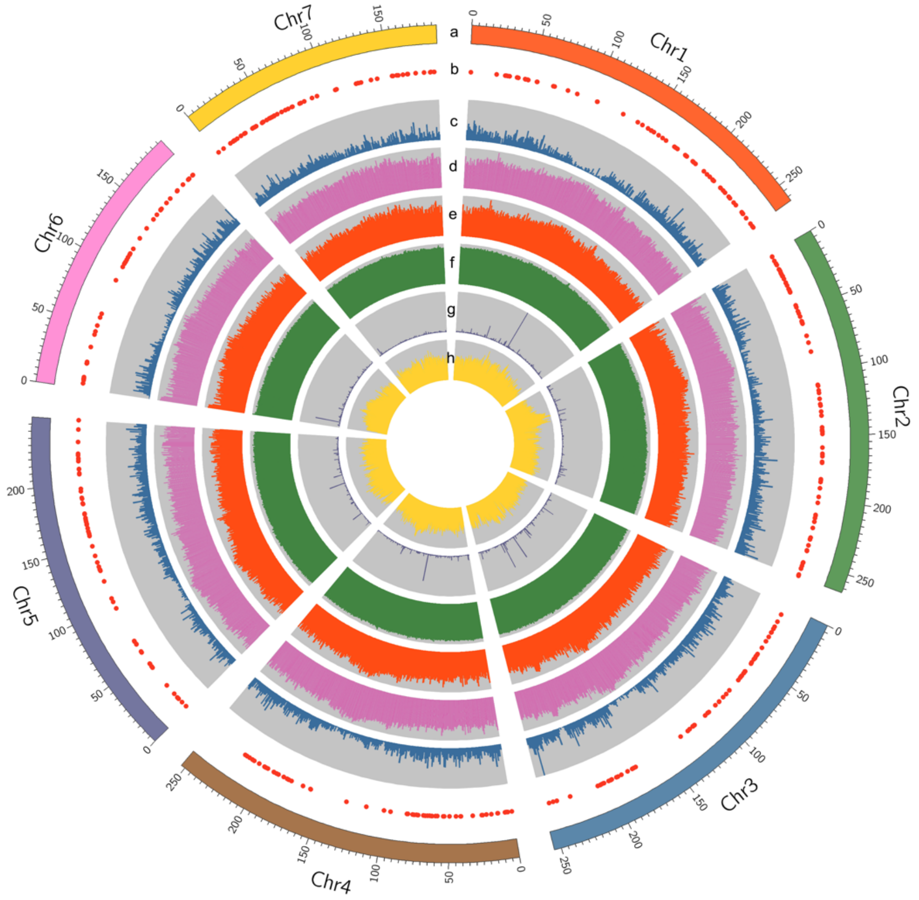

Supplementary Fig. 1 The orchardgrass genome landscape. Track a represents the seven chromosomes in Mb scale. Track b represents chromosomal distribution of 603 orthologs of *A. thaliana* flowering genes. Track c represents chromosomal distribution of gene models that were annotated, where gene density ranged from 371 bp/Mb to 380,434 bp/Mb. Track d represents the chromosomal distribution of repeat sequences where density of the repeat sequences was 74,261 bp/Mb to 903,485 bp/Mb. Track e represents chromosomal distribution of TE density, and TE density axis range was 74,261 bp/Mb – 895,797 bp/Mb. Track f represents the GC content along the assembled genome, which ranged between 41.0989%/Mb to 48.0363%/Mb. Track g represents the pooled gene expression level of five tissues (root, stem, leaf, flower, and spike). Track h represents chromosomal distribution of SNPs identified from 54 wild germplasm accessions, 11 cultivars and 11 unknown orchardgrass collection samples. The range of SNP density was 3,034/Mb – 111,599/Mb.

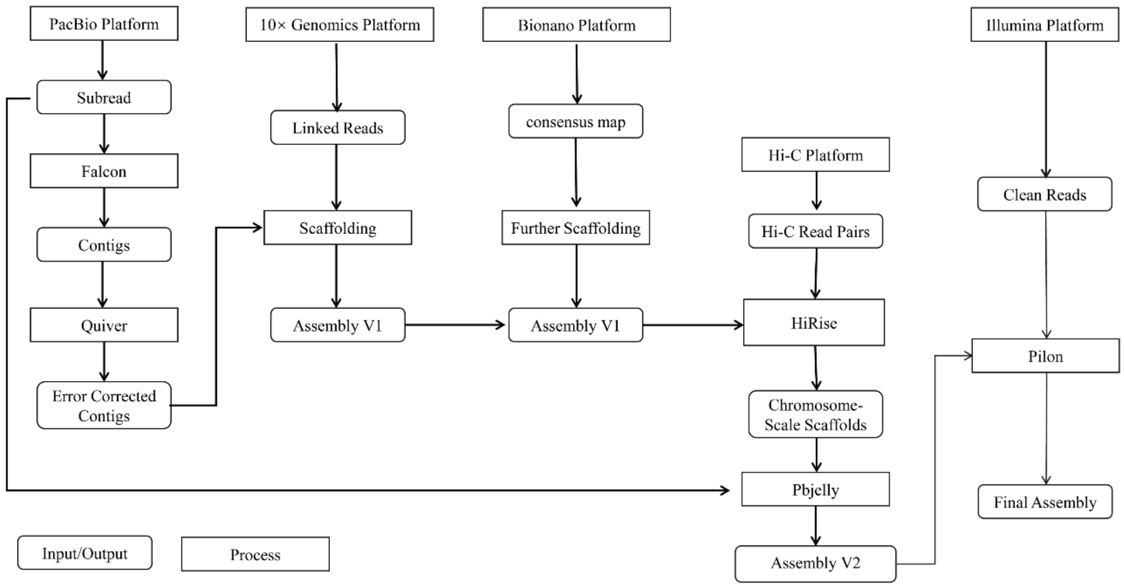

Supplementary Fig. 2 The workflow of orchardgrass genome assembling

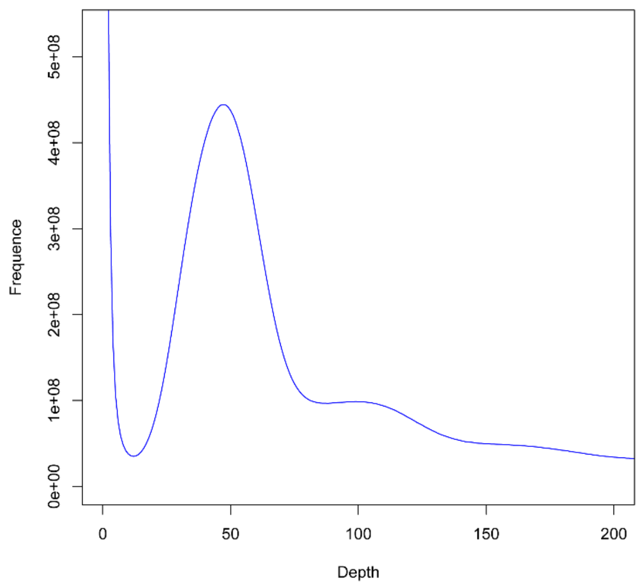

Supplementary Fig. 3 K-mer frequency distributions in orchardgrass. axis means sequence depth (X), and y axis means frequency of K-mer.

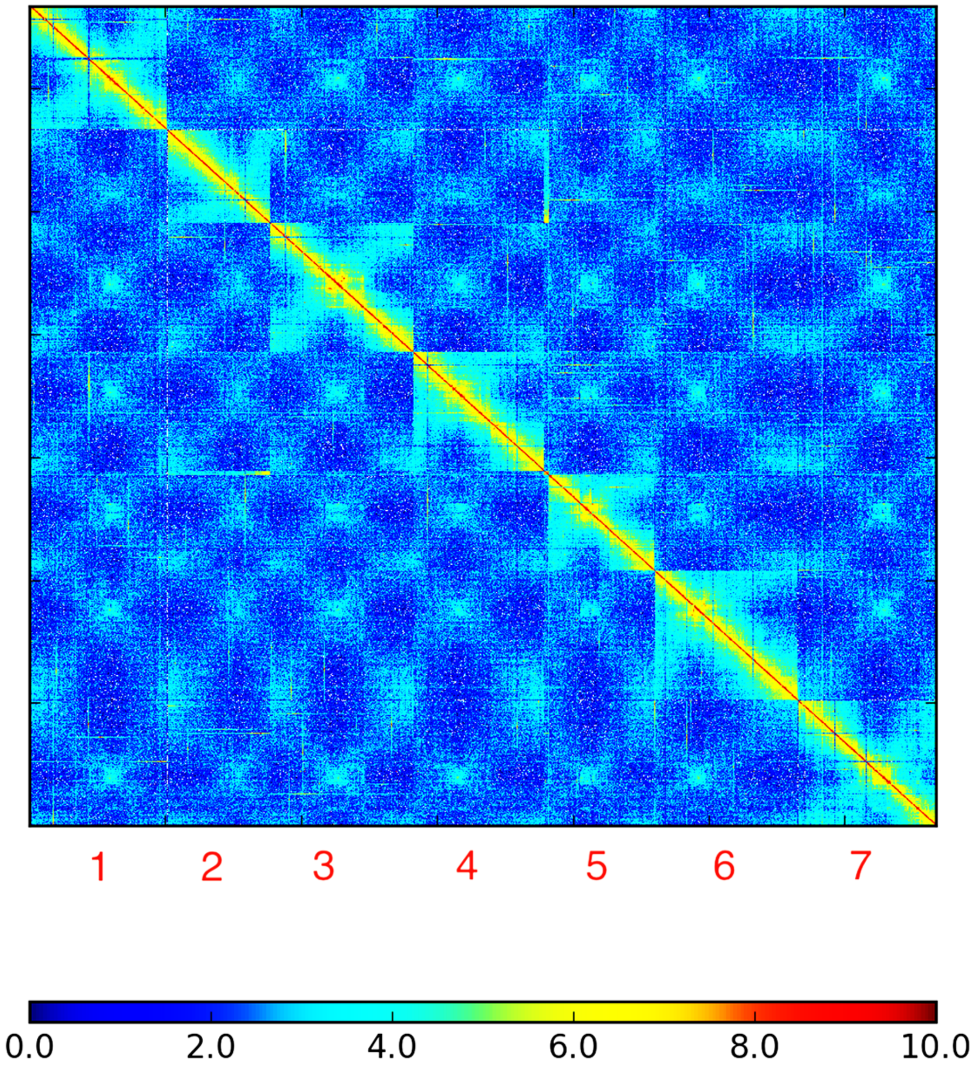

Supplementary Fig. 4 Scaffold Hi-C contact map data analysis. the x and y axes indicate the mapping positions of the first and second read in the read pair respectively, grouped into bins. The color of each square gives the number of read pairs within that bin. White vertical and black horizontal lines have been added to show the borders between scaffolds. Scaffolds less than 1 Mb are excluded.

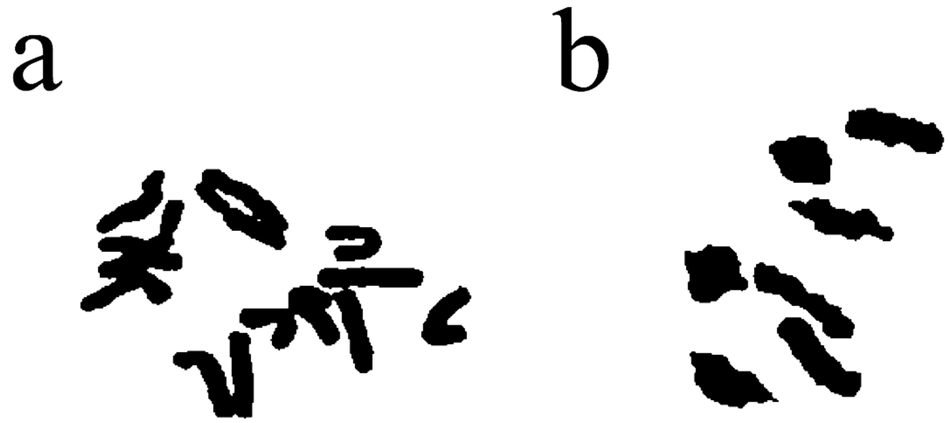

Supplementary Fig. 5 The chromosome number of the diplod orchardgrass (genotype 2006-1). a) seven pairs of sister chromosomes (Prometaphase, mitosis) b) seven pairs of sister chromosomes (Metaphase, mitosis).

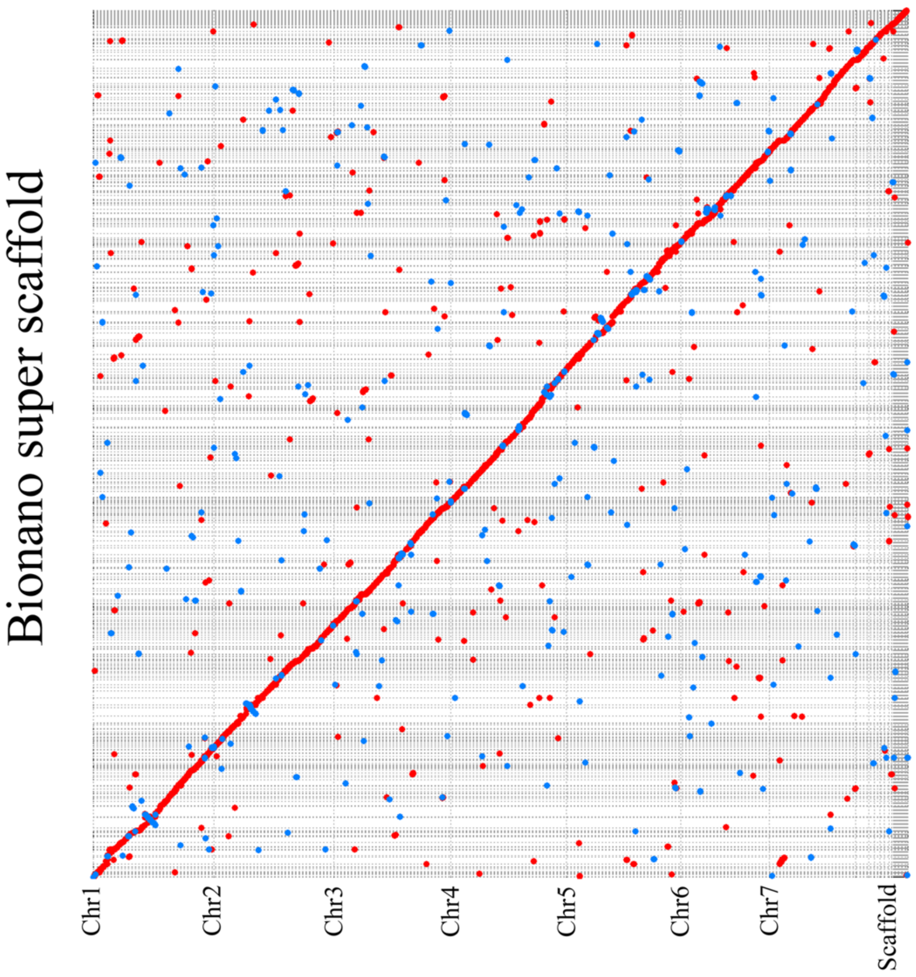

Supplementary Fig. 6 Consistency between Hi-C and BioNano. Blue dots mean negative direction, and red dots mean positive direction.

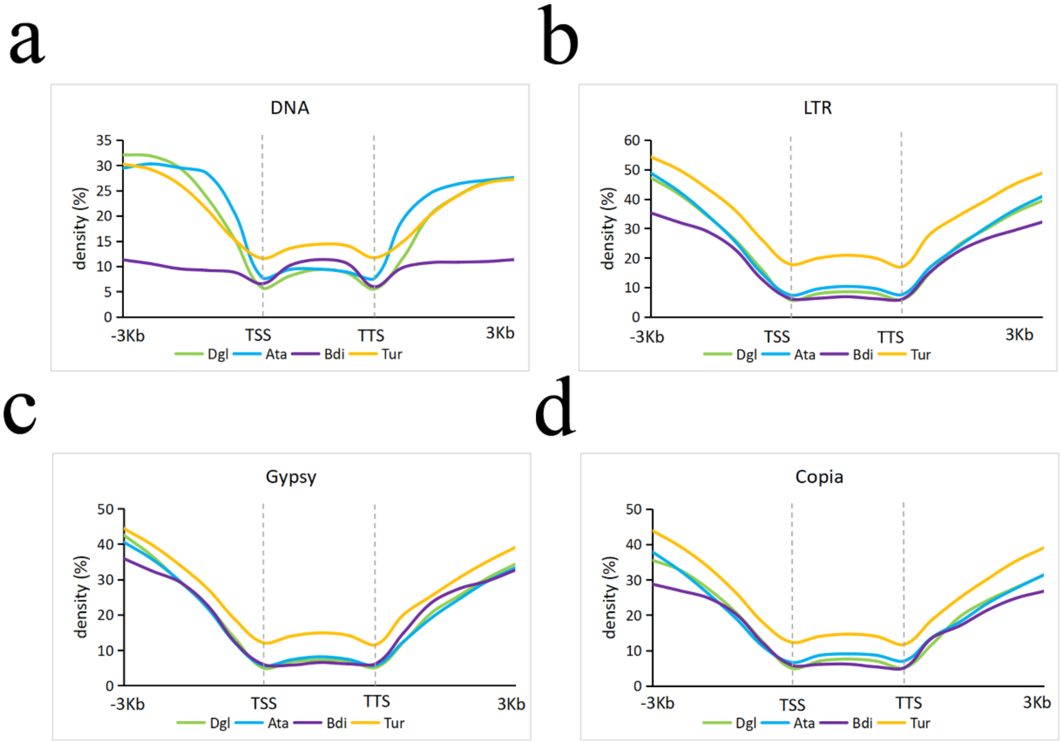

dSupplementary Fig. 7 The density of TEs surrounding genes. The density was determined to proportion of genes inserted by TEs in one locus to all genes inserted by TEs. TSS means transcript start point, and TTS means transcript end point. Dgl means orchardgrass, Ata means *A. tauschii*, Bdi means *B. distachyon*, and Tur means *T. Urartu*.

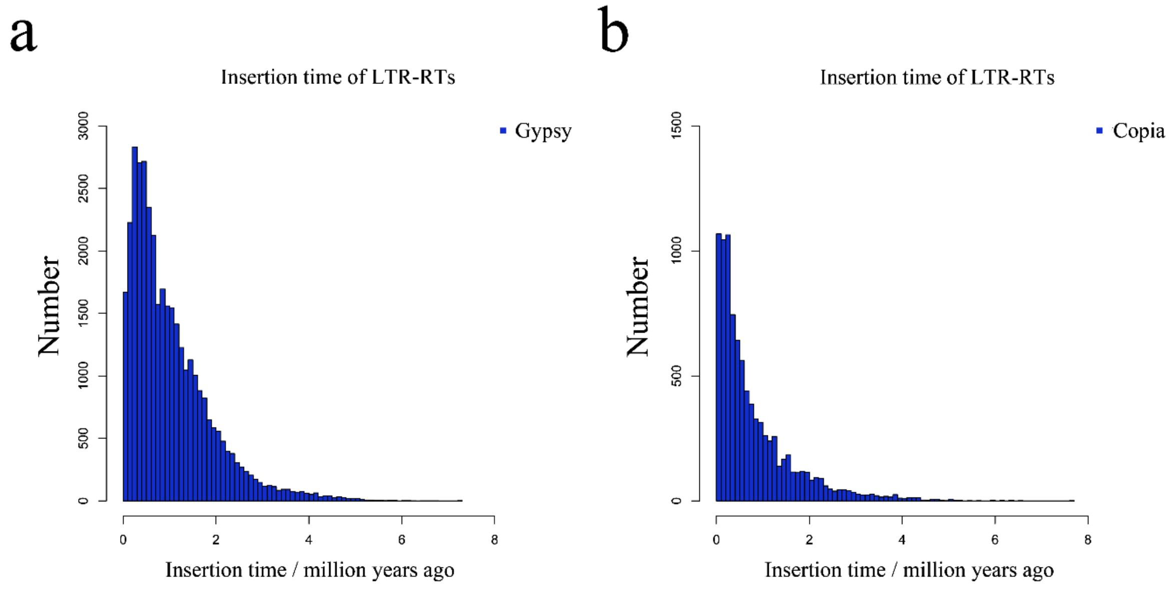

Supplementary Fig. 8 The distribution of desertion time for LTRs/Gypsy (a) and LTRs/Copia (b) for orchardgrass.

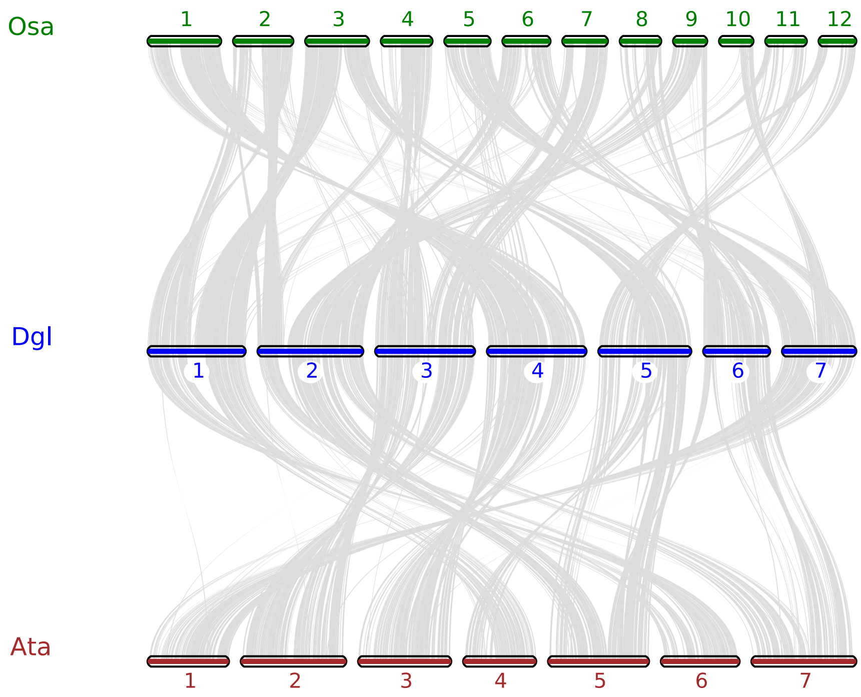

Supplementary Fig. 9 Synteny analysis of seven chromosomes from orchardgrass (Dgl) to twelve chromosomes from *Oryza sativa* (Osa) and seven chromosomes from *Aegilops tauschii* (Ata).

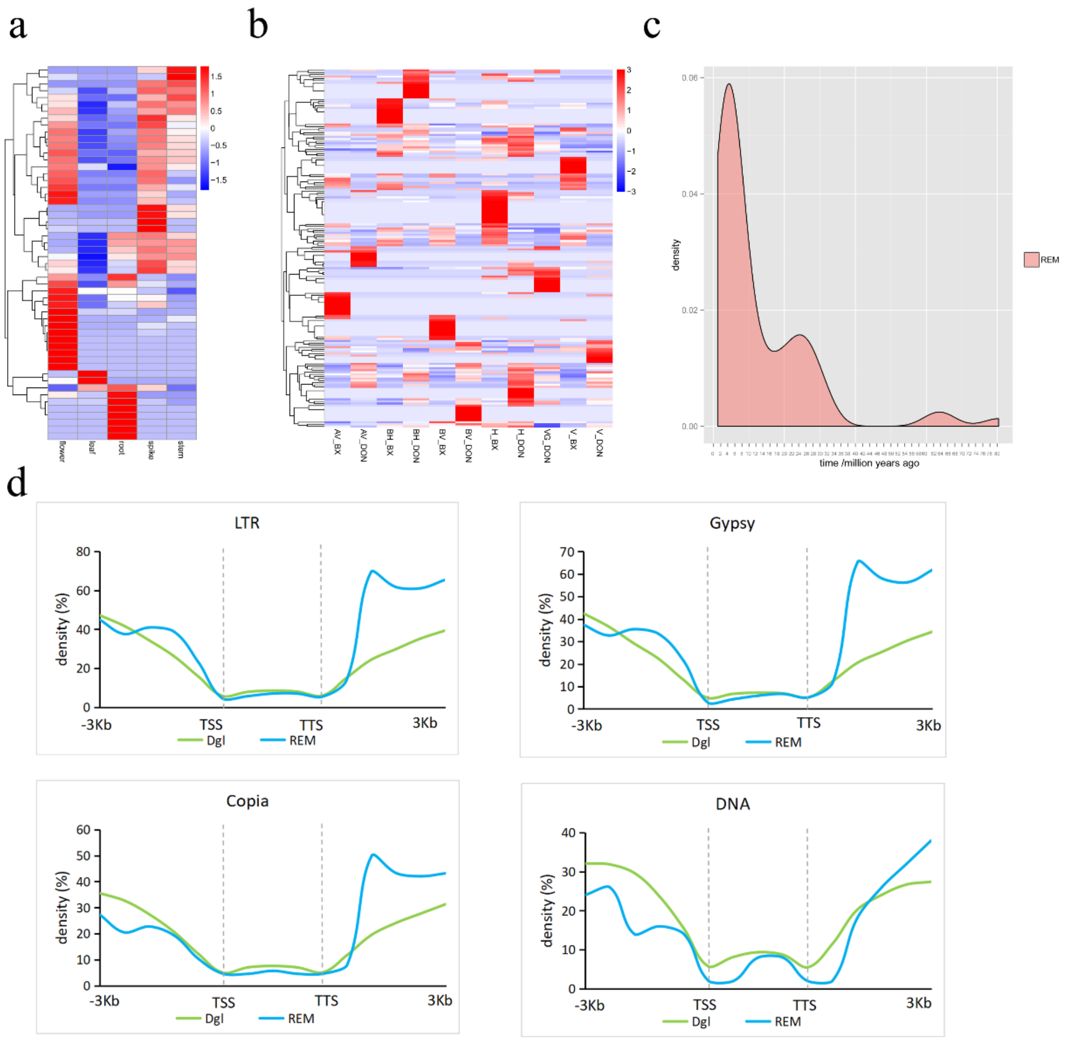

Supplementary Fig. 10 **REM family in orchardgrass.** (a) expression levels of REMs in different tissues. (b) expression levels of REMs in five flowering stages (BV, before vernalization; VE, vernalization; AV, after vernalization; VG, vegetative growth; BH, before heading, and HT, heading stage. Y axis represents the relative expression and x axis represents the different stages. BX indicates orchardgrass cultivar BAOXING, and DON indicates orchardgrass cultivar DONATA). (c) expansion time of REMs. (d) the density of transposons around REMs and all genes in orchardgrass. The density was determined to proportion of genes inserted by TEs in one locus to all genes inserted by TEs. TSS means transcript start point, and TTS means transcript end point.

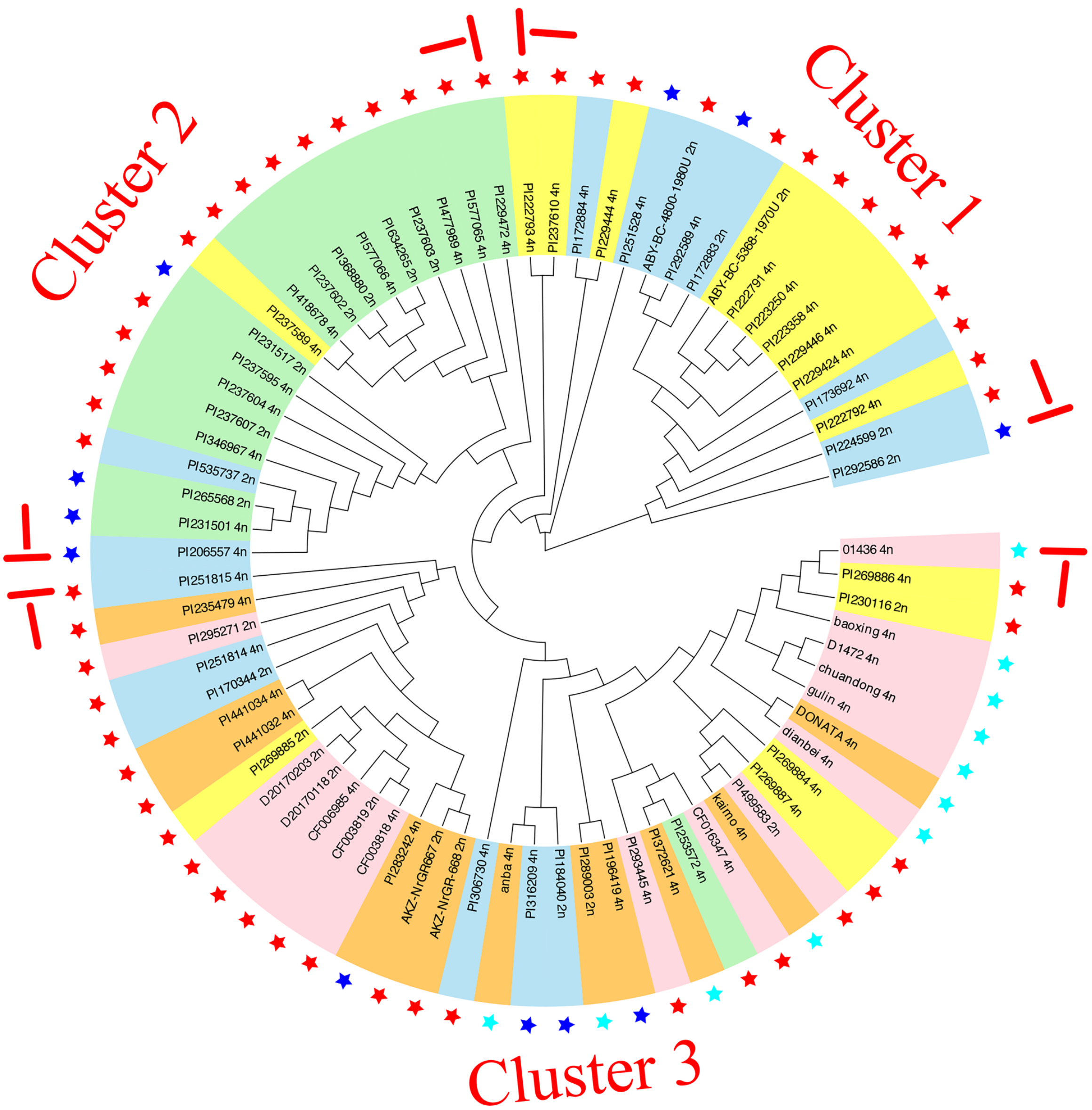

Supplementary Fig. 11 Phylogenetic tree of 76 orchardgrass accessions. Colors of inner circle indicate the materials come from different regions: Blue, East of Mediterranean; Yellow, Central Asia; Pink, East Asia; Green, West of Mediterranean; Orange, Northern Europe. Colors of outer circle indicate the wild (red), cultivar (light blue) and uncertain (dark blue) orchardgrass accessions.

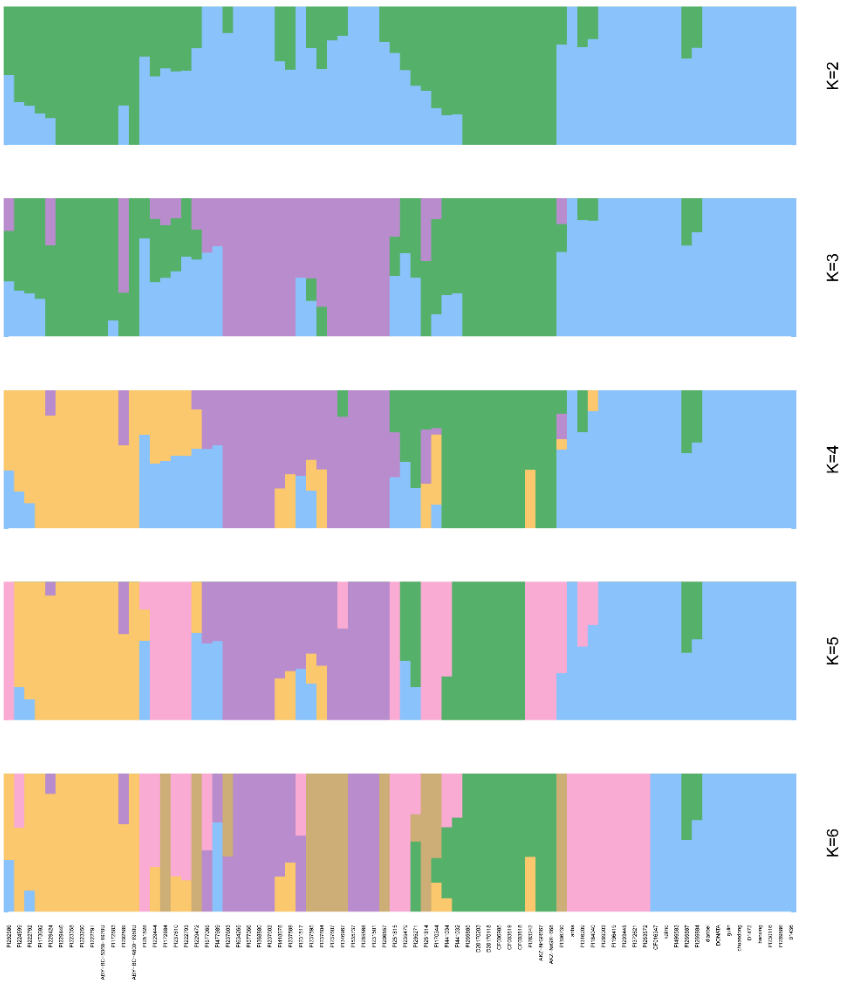

Supplementary Fig. 12 Structure analysis with different number of K value (k=2,3,4,5,6) of 76 orchardgrass.

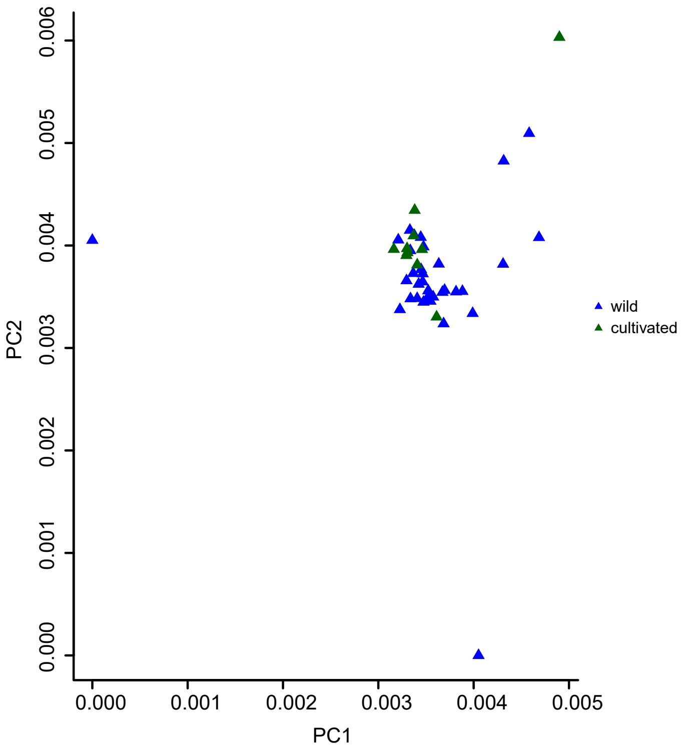

Supplementary Fig. 13 PCA plot of the first two components (PC1 and PC2) of 43 autotetraploid orchardgrass. blue triangles indicate wild accessions, and green triangles indicate cultivated accessions in orchardgrass.

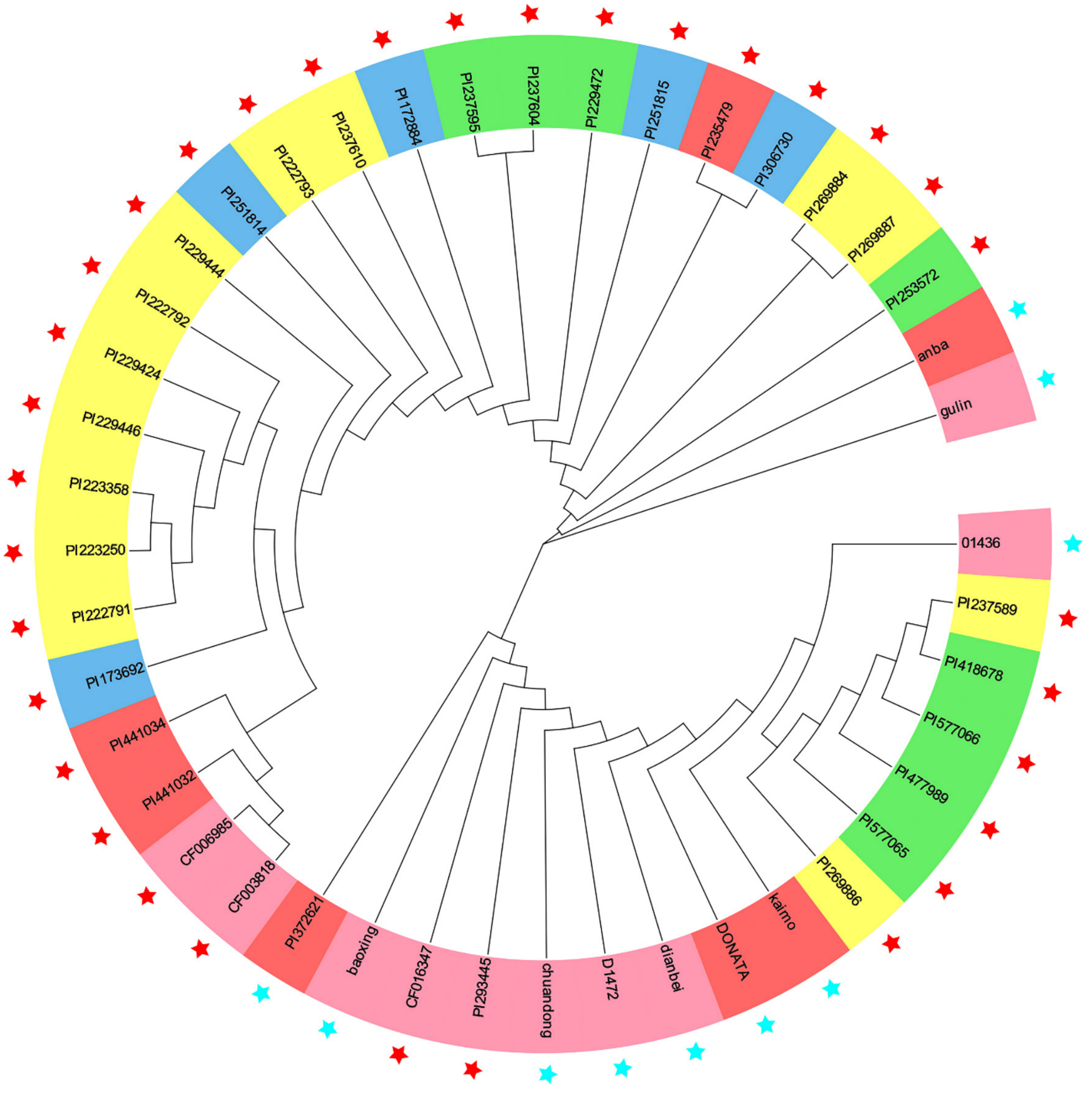

Supplementary Fig. 14 Phylogenetic tree of 43 autotetraploid orchardgrass. Colors of inner circle indicate the materials come from different regions: Blue, East of Mediterranean; Yellow, Central Asia; Pink, East Asia; Green, West of Mediterranean; Red, Northern Europe. Colors of outer circle indicate the wild (dark green) and cultivar (light green) orchardgrass accessions.

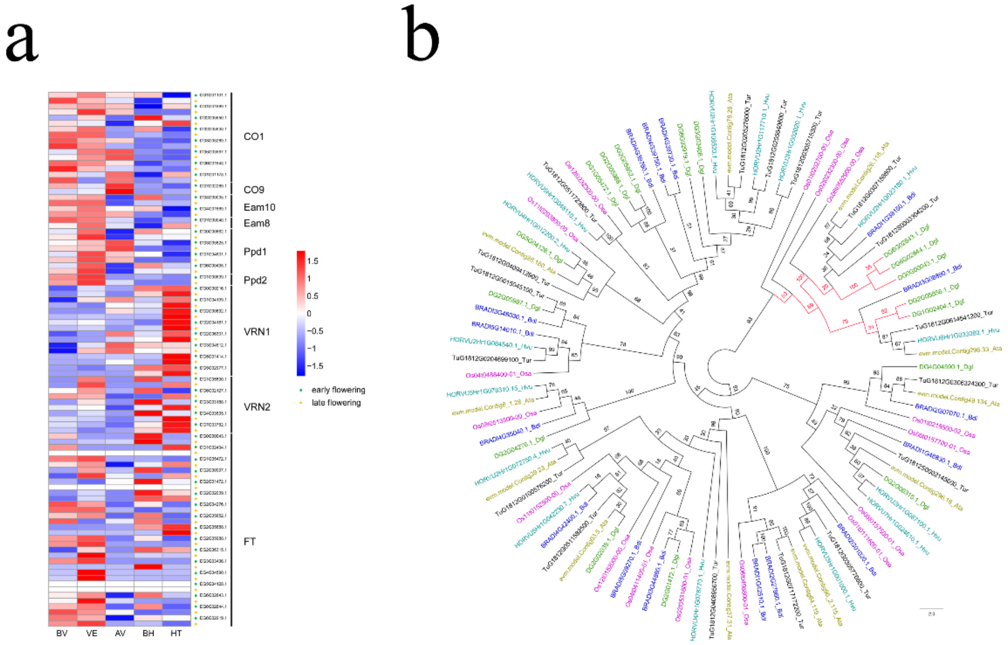

Supplementary Fig. 15 Analysis of important flowering related orthologues in orchardgrass. (a) Expression levels of several key orthologues associating with flowering time (BV, before vernalization; VE, vernalization; AV, after vernalization; VG, vegetative growth; BH, before heading, and HT, heading stage). (b) Phylogenetic tree of FT orthologues in orchardgrass, rice, *B. distachyon*, *H. vulgare*, *A. tauschii*, and *T. urartu*. Red line indicates five FT orthologues experience expansion during orchardgrass evolution.

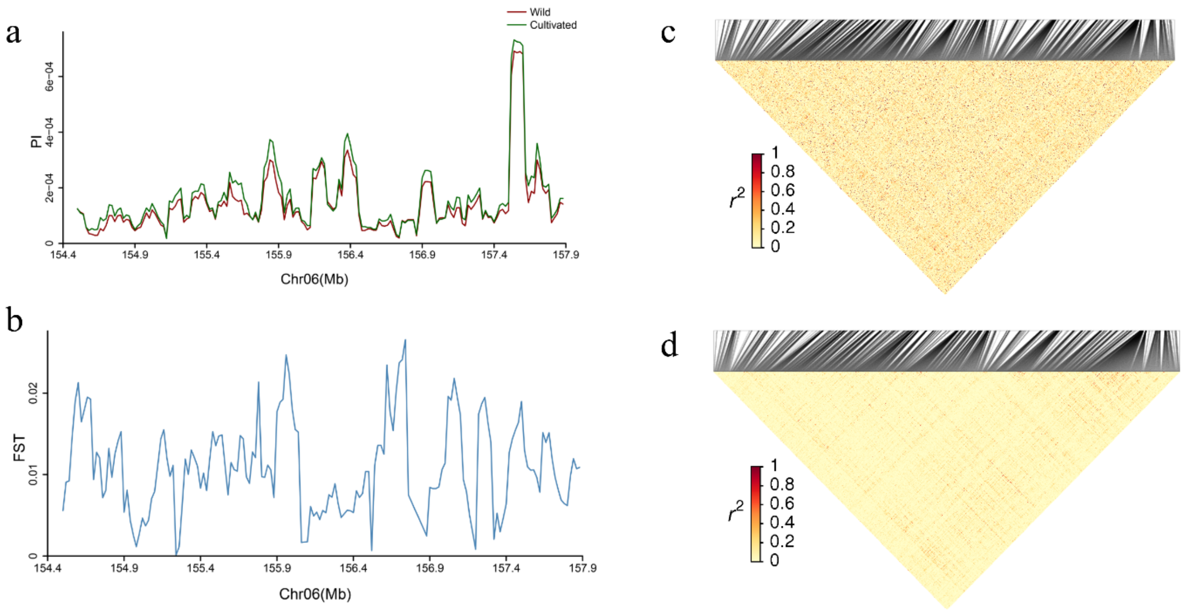

Supplementary Fig. 16 Nucleotide diversity (π) estimated in wild (red) and cultivated (green) orchardgrass (a) and the *FST* value (b), and patterns of LD in cultivated (c) and wild (d) in the 4.426 Mb region of orchardgrass chromosome 06.

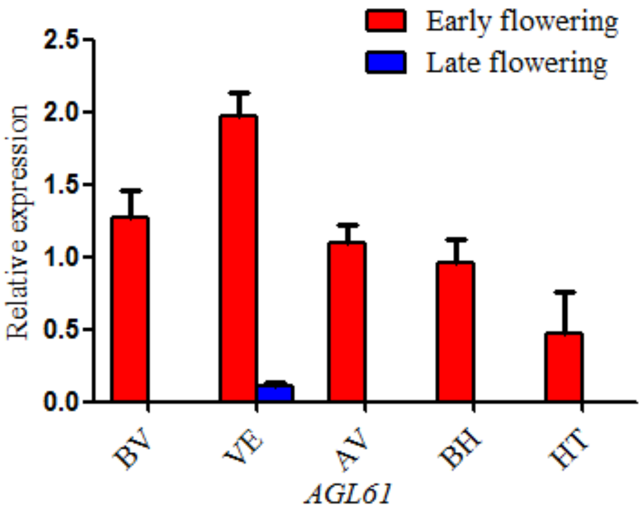

Supplementary Fig. 17 Comparison of *AGL61* expression during the five development stages in Orchardgrass. Error bars indicate ±s.d.; n = 3 biological replicates.

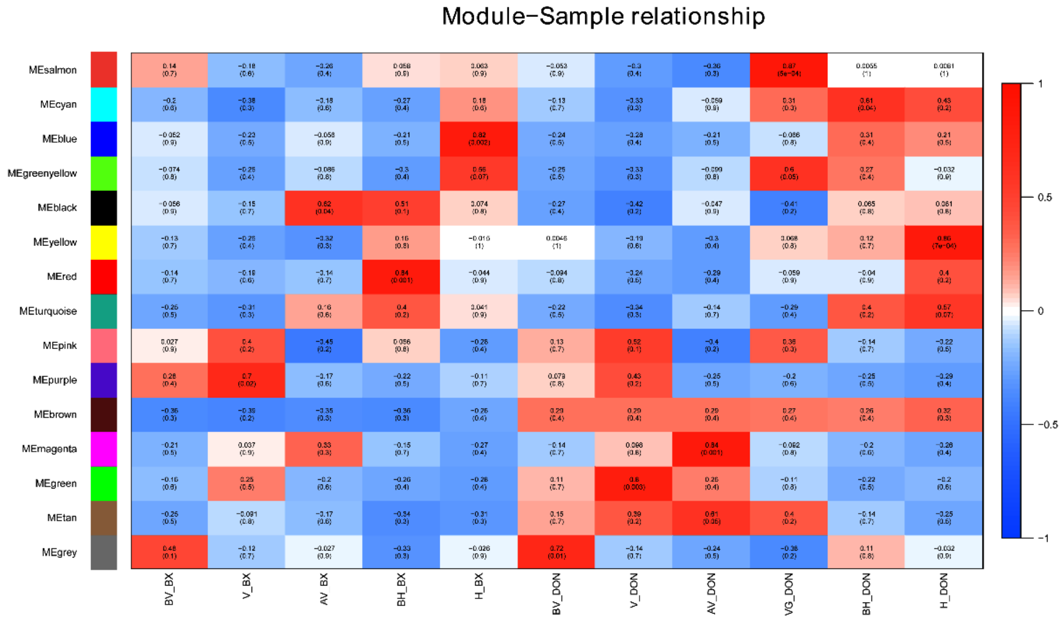

Supplementary Fig. 18 Module-Sample relationship. Heatmap shows the correlation between modules and samples, and the deeper red color represents the higher correlation.

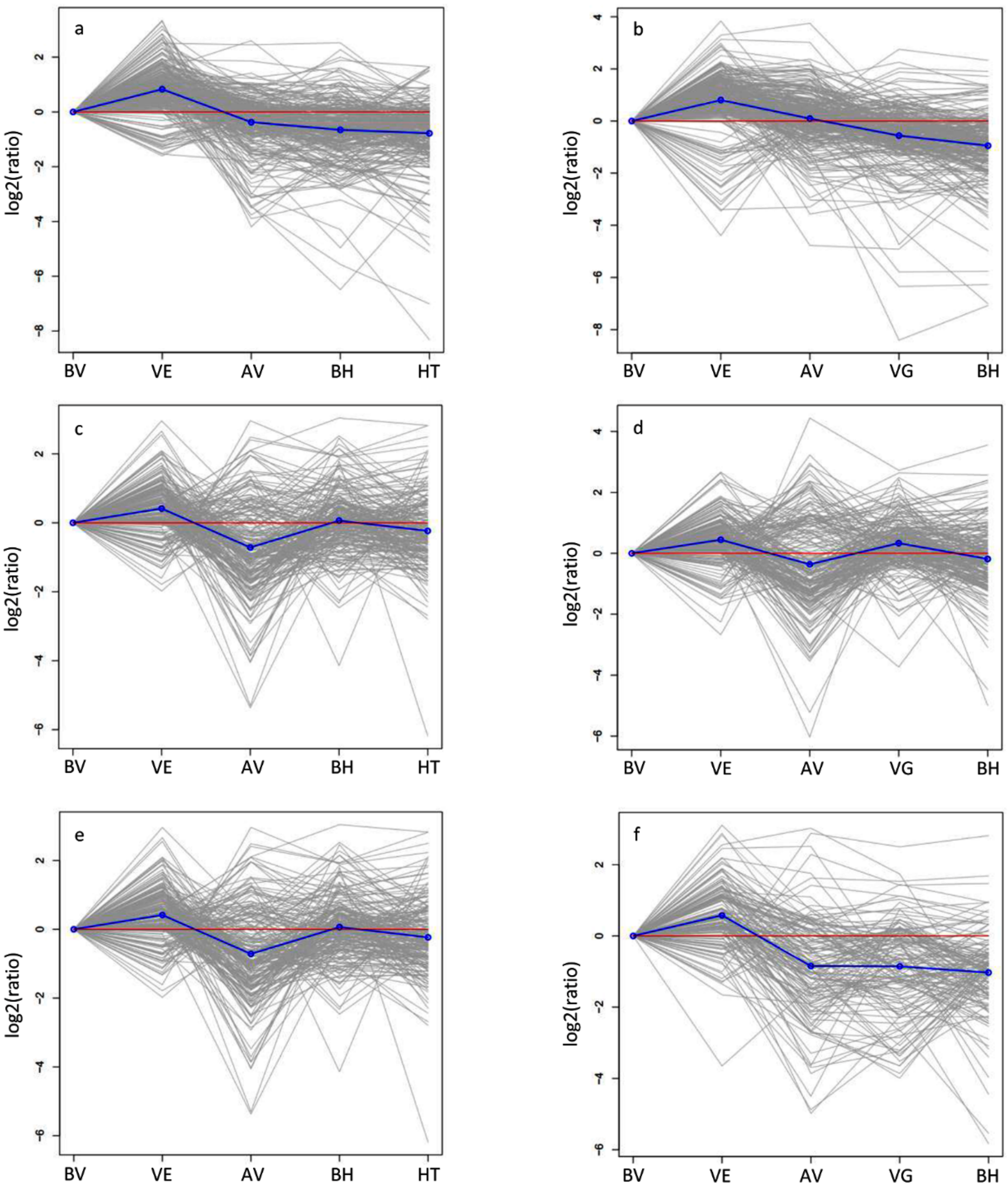

Supplementary Fig. 19 Expression pattern of genes in green, pink and purple module. (a) and (b) indicated the genes of green module in early and late flowering phenotype, (c) and (d) indicated the genes of pink module in early and late flowering phenotype, and (e) and (f) indicated the genes of purple module in early and late flowering phenotype.
