# Supplementary material for: Genome assembly provides insights into the genome evolution and flowering regulation of orchardgrass
Source: Plant Biotechnol J. 2019 Jul 30;18(2):373–88. doi: 10.1111/pbi.13205 (PMC6953241; doi:10.1111/pbi.13205)
Supplement: Supplementary file 3 — Appendix S1 Supplementary note. [file PBI-18-373-s003.docx]

**SUPPLEMENTARY Appendix S1**

**Supplementary Note Content**

**Supplementary Note.pdf: This document includes the following:**

**- 5 sections**

**- 19 supplementary figures**

**- 42 supplementary tables**

[1.Sequencing and genome assembly 2](#_Toc525327992)

[1.1 Plant materials 2](#_Toc525327993)

[1.2 DNA extraction and Illumina library preparation 2](#_Toc525327994)

[1.3 PacBio library construction and sequencing 3](#_Toc525327995)

[1.4 10X Genomics library construction and sequencing 3](#_Toc525327996)

[1.5 RNA extraction and library preparation 3](#_Toc525327997)

[1.6 Estimation of genome size and polyploidy 4](#_Toc525327998)

[1.7 Genome assembly 5](#_Toc525327999)

[1.8 Dovetail HiC library preparation and sequencing (multiple libraries) 5](#_Toc525328000)

[1.9 Scaffolding the assembly with HiRise 6](#_Toc525328001)

[1.10 SNP detection between two haplotypes 6](#_Toc525328002)

[1.11 Validation of the genome assembly 7](#_Toc525328003)

[2. Genome characterization and annotation 8](#_Toc525328004)

[2.1 Repetitive sequence annotation 8](#_Toc525328005)

[2.2 Identification of protein-coding genes 8](#_Toc525328006)

[2.3 Functional annotation of protein-coding genes 10](#_Toc525328007)

[3. Genome evolution 10](#_Toc525328008)

[3.1 Phylogenetic analysis 10](#_Toc525328009)

[3.2 Expansion and contraction of gene families 12](#_Toc525328010)

[3.3 Genome synteny and whole-genome duplication 12](#_Toc525328011)

[4. Resequencing 12](#_Toc525328012)

[4.1 Plant materials 12](#_Toc525328013)

[4.2 Library preparation and genome sequencing 13](#_Toc525328014)

[4.3 Read mapping and SNP/InDel calling 14](#_Toc525328015)

[4.4 Functional annotation of genetic variants 14](#_Toc525328016)

[4.5 Phylogenetic tree and population structure 15](#_Toc525328017)

[5. Candidate genes controlling the flowering time of orchardgrass 15](#_Toc525328018)

[5.1 Identification of flowering-time genes 15](#_Toc525328019)

[5.2 Vernalization transcriptome materials 15](#_Toc525328020)

[5.3 Transcriptome analysis 16](#_Toc525328021)

[5.4 BSA-sequencing materials 17](#_Toc525328022)

[5.5 BSA 17](#_Toc525328023)

[5.6 WGCNA 18](#_Toc525328024)

## 1. Sequencing and genome assembly

### 1.1 Plant materials

The diploid orchardgrass (*Dactylis glomerata* L.) accession 2006-1 (2n = 14) was used for genome sequencing. Accession 2006-1 is an elite wild accession collected from Wuxi, Chongqing, China (altitude: 2,475 m, 31°35.086′N, 109°0.84′E), and planted at the experimental station of Sichuan Agriculture University (30°42′N, 103°51′E; Wenjiang, Chengdu; annual mean temperature: 16.0℃, annual mean precipitation: 865.9 mm, and annual mean sunshine duration: 991.1 hours). The chromosome number of 2006-1 was counted under a microscope by squashing young root tips [[1](#_ENREF_1)] (Figure S5). A seven-day dark treatment was applied before leaf tissue sampling for Hi-C sequencing.

###

### 1.2 DNA extraction and Illumina library preparation

Genomic DNA was extracted from young leaf tissue of *D. glomerata* using a DNAsecure Plant Kit (TIANGEN, Beijing, China). Sequencing libraries with insert sizes ranging from 250 bp to 350 bp were constructed using a library construction kit according to the manufacturer’s instructions (Illumina, San Diego, CA). These two libraries were then sequenced using an Illumina HiSeq X Ten platform. The 382 Gb of raw reads generated was filtered according to sequencing quality, the presence of adaptor contaminants, and duplication. Quality control involved the following steps: (1) removing reads with >= 10% unidentified nucleotides (Ns); (2) removing reads with > 20% of bases having a Phred quality < 5; (3) removing reads with >10 nt aligned to the adapter, allowing <= 10% mismatches; and (4) removing putative PCR duplicates generated by PCR amplification during the library construction process (i.e., read 1 and 2 of two paired-end reads that were completely identical). Only high-quality reads were used for genome assembly.

###

### 1.3 PacBio library construction and sequencing

For 20-kb-insert-size library construction, at least 10 μg of sheared DNA is required. SMRTbell template preparation involved DNA concentration, damage repair, end repair, hairpin adapter ligation, and template purification. Finally, we carried out 20-kb single-molecule real-time (SMRT) DNA sequencing by PacBio and sequenced the DNA library on the PacBio Sequel platform, yielding approximately 167 Gb of PacBio data (Table S2).

###

### 1.4 10X Genomics library construction and sequencing

DNA sample preparation, indexing, and barcoding were carried out using a GemCode Instrument from 10X Genomics. A DNA sample of 1 ng with a length of 50 kb was used for the GEM reaction procedure during PCR, and 16-bp barcodes were introduced into droplets. Then, the droplets were fractured following the purification of the intermediate DNA library. The library was finally sequenced on an Illumina HiSeq X Ten instrument.

###

### 1.5 RNA extraction and library preparation

RNA-seq experiments were conducted for five types of tissue, namely, root, leaf, stem, flower, and spike, from the genotype 2006-1. Total RNA was extracted from the five types of tissue using an RNAprep Pure Plant Kit (TIANGEN, Beijing, China), and genomic DNA contaminants were removed using RNase-Free DNase I (TIANGEN, Beijing, China). The integrity of RNA was evaluated on a 1.0% agarose gel stained with ethidium bromide (EB), and its quality and quantity were assessed by using an Agilent 2100 Bioanalyzer (Agilent Technologies, CA, USA). Then, the integrated RNA was used for cDNA library construction and Illumina sequencing. The cDNA library was constructed using an NEBNext Ultra RNA Library Prep Kit for Illumina (NEB) following the manufacturer’s recommendations. Prepared libraries were sequenced on the Illumina HiSeq X Ten platform, generating 150-bp paired-end reads.

A single Iso-Seq library was also built from pooled RNA samples of the five different tissues for full-length transcriptome sequencing by PacBio technology. The RNA was synthesized to cDNA and subsequently amplified to generate double-stranded cDNA using a Clontech SMARTer PCR cDNA Synthesis Kit. The library construction and subsequent sequencing were described previously [[2](#_ENREF_2)]. Three size-selected Iso-Seq libraries (i.e., 1-2k, 2-3k, and 3-6k) were constructed according to the manufacturer’s instructions (<https://pacbio.secure.force.com/SamplePrep>) [[3](#_ENREF_3)]. Finally, the Iso-Seq libraries were sequenced on the PacBio Sequel platform, yielding approximately 12.79 Gb of PacBio data.

###

### 1.6 Estimation of genome size and polyploidy

K-mer frequency analysis was used to estimate genome characteristics [[4](#_ENREF_4)]. The genome size of orchardgrass was calculated based on k-mer (k = 17) statistics, using the modified Lander-Waterman algorithm. The total length of the sequence reads was divided by the sequencing depth and the peak value of the frequency curve representing the overall sequencing depth. We estimated the genome size with the following formula: N×(L-K+1)-B)/D=G, where N is the total number of sequence reads, L is the average length of sequence reads, K is the K-mer length (17 bp) [[5](#_ENREF_5)], B is the total number of low-frequency K-mers (frequency ≤ 1 in this analysis), G is the genome size, and D is the overall depth, estimated via the K-mer distribution. Heterozygosity was reflected by distributions of the number of distinct k-mers (k = 17). Flow cytometry was used to confirm the genome size of 2006-1 following a previously reported method [6], using a BD-FACSCalibur flow cytometer (BD, USA) and fluorochrome propidium iodide (PI).

We generated approximately 200-fold coverage of Illumina paired-end reads. With 50-fold coverage of those reads, we calculated k-mer frequency distributions and estimated that the orchardgrass genome size was 1.94 Gb (Figure S2; Table S1). The genome size of orchardgrass was estimated to be approximately 1.939 Gb by using flow cytometry, which was similar to the size estimated by the k-mer analysis (1.94 Gb; Table S1).

###

### 1.7 Genome assembly

*De novo* assembly of the long reads from the PacBio SMRT Sequencer was performed using FALCON ([*https://github.com/PacificBiosciences/FALCON/*](https://github.com/PacificBiosciences/FALCON/)) and FALCON-Unzip [[7](#_ENREF_7)]. To obtain enough corrected reads, the longest 55X coverage of subreads were firstly selected as seed reads to correct sequence errors. The corrected read N50 and coverage were 11.5K and 39X, respectively. Then, error-corrected reads were aligned to each other and assembled into genomic contigs using FALCON with the following parameters: length_cutoff_pr = 5,000, max_diff = 120, and max_cov = 130. After the initial assembly, FALCON-Unzip was used to produce [primary contigs](http://pb-falcon.readthedocs.io/en/latest/glossary.html#term-primary-contig) (p-contigs), which were then polished using Quiver [[8](#_ENREF_8)], which yielded an assembly with a contig N50 size of 1.05 Mb. The total length of this assembly version was 1,775.05 Mb. Then, we used BWA-MEM to align the 10X Genomics data to the assembly using default settings [[9](#_ENREF_9)]. Scaffolding was performed by FragScaff with the barcoded sequencing reads [[10](#_ENREF_10)], generating a genome with a scaffold N50 size of 3.41 Mb. The total length of this assembly version was 1,781.32 Mb, containing 0.35% Ns.

###

### 1.8 Dovetail Hi-C library preparation and sequencing (multiple libraries)

Two Dovetail Hi-C libraries were prepared in a similar manner as those described previously [[11](#_ENREF_11)]. Briefly, for each library, chromatin was fixed with formaldehyde in the nucleus and then extracted. The fixed chromatin was digested with DpnII, the 5’ overhangs were filled in with biotinylated nucleotides, and free blunt ends were ligated. After ligation, crosslinks were reversed, and the DNA was purified from protein. Purified DNA was treated via incubation with proteinase K at 65°C to remove biotin that was not internal to ligated fragments. The DNA was then sheared into an average fragment size of ~350 bp for library construction using NEBNext Ultra enzymes and Illumina-compatible adapters. Biotin-containing fragments were isolated using streptavidin beads before PCR enrichment of each library. The constructed libraries were sequenced on an Illumina HiSeq platform. The number and length of read pairs produced for each library were 237 million and 2x150 bp for library 1 and 249 million and and 2x150 bp for library 2, respectively. Together, these Dovetail Hi-C library reads provided 1,116X physical coverage of the genome (1-50 kb pairs).

###

### 1.9 Scaffolding the assembly with HiRise

The input *de novo* assembly and Dovetail Hi-C library reads were used as input data for HiRise, a software pipeline designed specifically for using proximity ligation data to scaffold genome assemblies [[12](#_ENREF_12)]. Dovetail Hi-C library sequences were aligned to the draft input assembly using a modified SNAP read mapper ([*http://snap.cs.berkeley.edu*](http://snap.cs.berkeley.edu)) [[13](#_ENREF_13)]. The separations of Dovetail Hi-C read pairs mapped onto draft scaffolds were analysed by HiRise to produce a likelihood model for genomic distance between read pairs, and the model was used to identify and break putative mis-joins, to score prospective joins, and to select joins above a threshold. Then, the Hi-C data were used for scaffolding by HiRise software, and 93.54% of sequences were grouped into seven super-scaffolds. Then, PBJelly software (*http://www.winsite.com/Home-Education/Science/PBJelly/*) was used to fill gaps within the PacBio data [[14](#_ENREF_14)]. Last, Pilon [[15](#_ENREF_15)] was used to perform error correction based on the Illumina sequences.

###

### 1.10 SNP detection between two haplotypes

The high-quality Illumina sequence reads were mapped to the genome assembly using the Burrows-Wheeler Aligner (BWA) with the command ‘mem -t 4 -k 32 –M’. The alignment results were converted to BAM files using SAMtools software. To reduce mismatches generated by PCR amplification before sequencing, duplicate reads were removed by SAMtools. After alignment, we performed SNP calling using a Bayesian approach as implemented in the package SAMtools, and the ‘mpileup’ command was used to identify SNPs with the parameters as ‘-q 1 -C 50 -S -D -m 2 -F 0.002 –u’. Finally, 595,453 single nucleotide polymorphisms (SNPs) were identified between the two haplotypes of the diploid, with 563,316 SNPs (94.6%) located in the intergenic regions and the remaining SNPs (5.4%) located in genic and upstream regions (Table S4).

###

### 1.11 Validation of the genome assembly

The Core Eukaryotic Genes Mapping Approach (CEGMA) (*http://korflab.ucdavis.edu/dataseda/cegma/*) [[16](#_ENREF_16)] pipeline was used to assess the completeness of the genome assembly or annotations. CEGMA analysis showed that the genome assembled completely covered 231 (93.15%) of the 248 core eukaryotic genes (CEGs) and partially covered 13 of the CEGs, indicating that less than 2% of the CEGs were not detected (Table S5).

Benchmarking Universal Single-Copy Orthologs (BUSCO, *http://busco.ezlab.org/*) [[17](#_ENREF_17)] provides quantitative measures of genome assembly, gene set, and transcriptome completeness based on evolutionarily informed expectations of gene content from near-universal single-copy orthologues selected from [OrthoDB v9](http://orthodb.org/). BUSCO analysis showed that 96.7% of the 1,440 plant single-copy orthologues were complete (Table S5).

The draft assembly was further evaluated by mapping the high-quality reads from short-insert-size PE libraries to the genome assembly using BWA-MEM. The distribution of the sequencing depth at each position was calculated using SAMtools to assess the completeness of the genome assembly [[18](#_ENREF_18)]. The mapping rate was 99.62%, and the genome coverage was 99.66% (Table S6). A total of 53,836 publicly available expressed sequence tag (EST) sequences of *D. glomerata* were downloaded from the NCBI and mapped to the genome using BLAT (*http://genome.ucsc.edu/goldenpath/help/blatSpec.html*) with an identity >95%, and 49,017 (91.05%) of them were mapped to the reference genome with more than 90% coverage (Table S7) [[19](#_ENREF_19)]. Collectively, these data indicated the high base accuracy and high genome coverage of the orchardgrass assembly sequence.

## 2. Genome characterization and annotation

### 2.1 Repetitive sequence annotation

Transposable elements (TEs) in the *D. glomerata* genome were identified by combining *de novo*-based and homology-based approaches. For the *de novo*-based approach, we used RepeatModeler [[21](#_ENREF_21)] ([*http://www.repeatmasker.org/RepeatModeler.html*](http://www.repeatmasker.org/RepeatModeler.html)), a *de novo* repeat family identification and modelling package, LTR_FINDER ([*http://tlife.fudan.edu.cn/ltr_finder/*](http://tlife.fudan.edu.cn/ltr_finder/)) [[22](#_ENREF_22)], and RepeatScout ([*http://www.repeatmasker.org/*](http://www.repeatmasker.org/)) [[23](#_ENREF_23)] to build a *de novo* repeat library. For the homology-based approach, we used RepeatMasker ([*http://www.repeatmasker.org*](http://www.repeatmasker.org), version 3.3.0) against the Repbase TE library and RepeatProteinMask ([*http://www.repeatmasker.org/*](http://www.repeatmasker.org/)) against the TE protein database [[24](#_ENREF_24)]. The results from repetitive sequence annotation above were combined, and the repetitive sequence content accounted for 68.22% of the *D. glomerata* genome.

###

### 2.2 Identification of protein-coding genes

To predict protein-coding genes in the *D. glomerata* genome, we used homology-based prediction, *de novo­* prediction and transcriptome-based prediction. Homologue proteins from four plant genomes (*Arabidopsis thaliana, Oryza sativa, Triticum aestivum* and *Zea mays*) were downloaded from Ensembl Plants (http://plants.ensembl.org/index.html). Protein sequences from these genomes were aligned to the *D. glomerata* genome assembly using tblastN [[25](#_ENREF_25)], with an E-value cut-off of 1e-5. The BLAST hits were conjoined by Solar software [[26](#_ENREF_26)]. GeneWise (<https://www.ebi.ac.uk/Tools/psa/genewise>) was used to predict the exact gene structure of the corresponding genomic regions in each BLAST hit (Homo-set) [[27](#_ENREF_27)]. For transcriptome-based prediction methods, RNA-seq data were mapped to the assembly using TopHat (<http://ccb.jhu>. edu/software/tophat/index.shtml, version 2.0.8) and Cufflinks (<http://cole-trapnell-lab.github.io/cufflinks/>, version 2.1.1) [[28](#_ENREF_28), [29](#_ENREF_29)]. In addition, PacBio RNA-seq data were used to create several pseudo-ESTs. These pseudo-ESTs were also mapped to the assembly, and gene models were predicted by PASA (<http://pasapipeline.github.io/>)[[30](#_ENREF_30)]. This gene set was denoted PASA-T-set and was used to train *ab initio* gene prediction programs. Five *ab initio* gene prediction programs, namely, Augustus (<http://augustus.gobics.de/>, version 2.5.5), GENSCAN (<http://genes.mit.edu/GENSCAN.html>, version 1.0), GlimmerHMM (<http://ccb.jhu.edu/software/glimmerhmm/>, version 3.0.1), geneid (<http://genome.crg.es/software/geneid/>), and SNAP (<http://korflab.ucdavis.edu/software.html>), were used to predict coding regions in the repeat-masked genome [[31-34](#_ENREF_31)]. Gene model evidence from Homo-set, Cufflinks-set, PASA-T-set and *ab initio* programs were combined by EVidenceModeler (EVM) (<http://evidencemodeler.sourceforge.net/>) into a non-redundant set of gene structures [[35](#_ENREF_35)]. Finally, a total of 40,088 genes were predicted from the *D. glomerata* genome.

###

### 2.3 Functional annotation of protein-coding genes

Functional annotation of protein-coding genes was achieved by using BLASTP (E-value: 1e-05) against two integrated protein sequence databases [[36](#_ENREF_36)]: SwissProt (<http://web.expasy.org/docs/swiss-prot_guideline.html>) and NR (ftp://ftp.ncbi.nih.gov/blast/db/). Protein domains were annotated by searching against the InterPro ((<http://www.ebi.ac.uk/interpro/>, V32.0) and Pfam (<http://pfam.xfam.org/>, V27.0) databases, using InterProScan (V4.8) and HMMER (<http://www.hmmer.org/>, V3.1), respectively [[37-40](#_ENREF_37)]. The Gene Ontology (GO, <http://www.geneontology.org/page/go-database>) terms for each gene were obtained from the corresponding InterPro or Pfam entry. The pathways in which the genes might be involved were assigned by BLAST against the Kyoto Encyclopedia of Genes and Genomes (KEGG) database (<http://www.kegg.jp/kegg/kegg1.html>, release 53), with an E-value cut-off of 1e-05. A total of 36,522 genes were predicted to be functional, accounting for 91.1% of all genes in the *D. glomerata* genome. Alternative splicing (AS) events were analysed with SpliceGrapher software [[41](#_ENREF_41)], and alternative polyadenylation (APA) events were identified with GMAP software (<http://research-pub.gene.com/gmap/>). We identified 10,977 genes with APA or AS (Table S40).

##

## 3. Genome evolution

### 3.1 Phylogenetic analysis

The protein sequences of *A. thaliana*, *Populus trichocarpa*, *O. sativa*, *Sorghum bicolor*, *Z. mays*, *Setaria italica*, *Brachypodium distachyon*, and *Musa acuminata* were downloaded from Phytozome 12 (<https://phytozome.jgi.doe.gov/pz/portal.html)>, and those from *Hordeum vulgare*, *Triticum urartu*, *Aegilops tauschii*, and *Elaeis guineensis* were downloaded from the NCBI (https://www.ncbi.nlm.nih.gov/). All the genes of the 13 species were filtered as follows: (a) when multiple transcripts were present in one gene, only the longest transcript in the coding region was taken for further analysis, and (b) the genes encoding proteins with fewer than 50 amino acids were filtered out. We obtained the similarities between the protein sequences of all species through BLASTP with an E-value < 1e-5. The protein sequences of all 13 species were clustered into paralogues and orthologues using the program OrthoMCL (http://orthomcl.org/orthomcl/) with an inflation parameter equal to 1.5. After gene family clustering, we aligned all 803 single-copy gene protein sequences by MUSCLE [[42](#_ENREF_42)] and combined all the alignment results into a super-alignment matrix. Then, a phylogenetic tree of the 13 species was constructed using RAxML (http://sco.h-its.org/exelixis/web/software/raxml/index.html) with the maximum likelihood method and 100 bootstrap replicates [[43](#_ENREF_43)]. *A. thaliana* and *P. trichocarpa* were used as outgroups in the phylogenetic tree. Finally, the MCMCTree program (http://abacus.gene.ucl.ac.uk/software/paml.html) implemented in Phylogenetic Analysis by Maximum Likelihood (PAML) was applied to infer divergence time based on the phylogenetic tree [[44](#_ENREF_44)]. The MCMCTree run parameters were a burn-in of 10,000, sample number of 100,000, and sample frequency of 2. The calibration time of divergence between *A. thaliana* and *O. sativa* was 120.0-155.8 Million years ago (Mya), *O. sativa* and *M. acuminata* was 105.0-124.7 Mya, *O. sativa* and *B. distachyon* was 39.4-53.8 Mya, *T. urartu* and *A. tauschii* was 3.2-5.3 Mya, *A. thaliana* and *P. trichocarpa* was 99.9-118.8 Mya, and *S. italica* and *S. bicolor* was 22.7-28.5 Mya, according to the TimeTree database (<http://www.timetree.org/>).

Positive selection analysis was conducted based on the protein sequences of the 4,311 single-copy gene families in the six Poaceae species within the same cluster group (Fig. 3a) (*D. glomerata*, *B. distachyon*, *H. vulgare*, *T. urartu*, *O. sativa*, and *A. tauschii*). Of these single-copy genes, 765 in orchardgrass underwent positive selection (*P* < 0.05) (Table S41-42).

###

### 3.2 Expansion and contraction of gene families

We determined the expansion and contraction of the gene families by comparing the cluster size differences between the ancestor and each species using the CAFÉ program [[45](#_ENREF_45)]. A random birth and death model was used to evaluate the changes in gene families along each lineage of the phylogenetic tree. A probabilistic graphical model (PGM) was introduced to calculate the probability of transitions in gene family size from parent to child nodes in the phylogeny. Using conditional likelihoods as the test statistics, we calculated the corresponding *p*-values in each lineage, and a *p*-value < 0.05 was used to identify families that had significantly expanded and contracted.

###

### 3.3 Genome synteny and whole-genome duplication

To identify syntenic blocks, the protein sequences from *D. glomerata, O. sativa*, and *B. distachyon* were searched against themselves using BLASTp (E-value<1e-5). The results were subjected to MCScanX (-a, -e:1e-5, -u:1, -s:5) to determine syntenic blocks [[46](#_ENREF_46)]. At least five genes were required to define synteny. Then, we constructed the number of synonymous substitutions per synonymous site (K) distribution for each gene pair from the aligned blocks to identify speciation or whole-genome duplication events that occurred during their evolutionary history. The synteny blocks between chromosomes were visualized by Circos (Krzywinski et l. 2009).

## 4. Resequencing

### 4.1 Plant materials

Young leaf tissue samples from 76 orchardgrass genotypes were collected for DNA re-sequencing (Table S28). Individuals (including 11 orchardgrass cultivars, 54 wild collections and 11 individuals with uncertain improvement status) were obtained from Sichuan Agricultural University (China), the National Plant Germplasm System (USA) and the United States Department of Agriculture (USA). These 76 accessions included 13 orchardgrass subspecies: *D. glomerata subsp. judaica* (1 individual), *D. glomerate subsp. parthiana* (1 individual), *D. glomerata subsp. woronowii* (1 individual), *D. glomerata subsp. himalayensis* (1 individual), *D. glomerata subsp. glomerata* (45 individuals), *D. glomerata subsp. juncinella* (1 individual), *D. glomerata subsp. lusitanica* (3 individuals), *D. glomerata subsp. hispanica* (8 individuals), *D. glomerata subsp. marina* (6 individuals), *D. glomerata subsp. smithii* (3 individual), *D. glomerata subsp. aschersoniana* (2 individual), *D. glomerata subsp. lobata* (3 individuals), *D. glomerata subsp. santai* (1 individual). These 76 accessions originated from Europe, the Mediterranean, Central Asia, India, and China and thus represented the rich genetic diversity and wide geographical distribution of orchardgrass (Table S28). Flow cytometry was used to confirm the ploidy levels of each individual, following the previously reported method (Doležel, Greilhuber et al. 2007) and using 2006-1 as a reference and a BD-FACSCalibur flow cytometer (BD, USA).

###

### 4.2 Library preparation and genome sequencing

Genomic DNA was extracted by using a DNAsecure Plant Kit (TIANGEN). Sequencing libraries were generated using a TruSeq Nano DNA HT Sample preparation Kit (Illumina, USA) following the manufacturer’s recommendations, and index codes were added to attribute sequences to each sample. These libraries were sequenced on the NovaSeq platform to obtain 150-bp paired-end reads. To obtain reliable reads, the raw reads were subjected to a series of quality control procedures to remove low-quality reads; the method of quality control was described earlier (1.2 DNA extraction and Illumina library preparation). Finally, we obtained 1712.94 Gb of high-quality paired-end reads (average 12.06-fold depth, 146X).

###

### 4.3 Read mapping and SNP/InDel calling

The high-quality paired-end reads were mapped to the *D. glomerata* reference genome using the BWA [[47](#_ENREF_47)] (with the command ‘mem -t 4 -k 32 –M’). The alignment results were converted to BAM files using SAMtools software [[18](#_ENREF_18)]. To reduce mismatches generated by PCR amplification before sequencing, duplicated reads were removed by SAMtools. After alignment, we performed SNP calling on a population scale using a Bayesian approach as implemented in the package SAMtools. We then calculated genotype likelihoods from reads for each individual at each genomic location and the allele frequencies in the population with a Bayesian approach. The ‘mpileup’ command was used to identify SNPs with the parameters ‘-q 1 -C 50 -S -D -m 2 -F 0.002 –u’. Only high-quality SNPs (coverage depth >= 6, root mean square (RMS) mapping quality >= 20, minor allele frequency (maf) >=0.01, and misses <= 0.2) were kept for subsequent analysis. InDel calling was similar to the SNP calling procedure described above.

###

### 4.4 Functional annotation of genetic variants

SNP/InDel annotation of the *D. glomerata* genome was performed using the package ANNOVAR (for functional annotation of genetic variants from high-throughput sequencing data). Based on the genome annotation, SNPs/InDels were categorized as being located in exonic regions (overlapping with a coding exon), splicing sites (within 2 bp of a splicing junction), 5' and 3' untranslated regions (UTRs), intronic regions (overlapping with an intron), upstream and downstream regions (within a 1-kb region upstream or downstream of the transcript start point (TSS) or transcript end point (TTS)), and intergenic regions. The SNPs in coding exons were further grouped into synonymous or nonsynonymous mutations. The SNPs causing gain of a stop codon, loss of a stop codon, or splicing were considered large-effect SNPs. We further classified InDels in coding exons as frameshift deletions, non-frameshift deletions, frameshift insertions and non-frameshift insertions.

### 4.5 Phylogenetic tree and population structure

To clarify the phylogenetic relationships from a genome-wide perspective, an individual-based neighbour-joining (NJ) tree was constructed using the software TreeBeST v1.9.2 [[48](#_ENREF_48)] with 1,000 bootstraps. Moreover, the population genetic structure was examined *via* the program Admixture 1.23 [[50](#_ENREF_50)], and the number of assumed genetic clusters (K) was set from 2 to 6, with 10,000 iterations for each run.

##

## 5. Candidate genes controlling the flowering time of orchardgrass

### 5.1 Identification of flowering-time genes

Flowering-time genes in *A. thaliana* were retrieved from a recently developed database, FLOR-ID, which includes 295 protein-coding genes and describes their interactions [[51](#_ENREF_51)]. Then, using *A. thaliana* homologues as queries, we identified the candidates in *D. glomerata* by BLASTP with an E-value of 1e-5. If the candidate genes were in a common family in OrthoMCL, they were predicted by PFAM (http://pfam.xfam.org/) [[38](#_ENREF_38), [52](#_ENREF_52)]. Only the candidate genes that had the same protein domain were further considered as potential candidate genes.

###

### 5.2 Vernalization transcriptome materials

To examine the transcriptional dynamics of the genes active during critical vernalization stages and the transcriptional differences between early and late flowering of orchardgrass, the two *D*. *glomerata* varieties “DONATA” (late flowering) and “BAOXING” (early flowering) were chosen for transcriptome analysis. The plant materials were grown in pots at Sichuan Agricultural University (30°42′N, 103°51′E; Wenjiang, Chengdu) under natural light conditions until December 2016. The plants were then transferred to a field in Chongzhou (30°37′N, 103°40′; Chongzhou, Chengdu) for vernalization induction from January 2016 to March 2016. Samples of five critical developmental stages were collected for analysis of the flowering transcriptome: January 4th (pre-vernalization), February 2nd (vernalization), March 2nd (post-vernalization), March 24th (cv. BAOXING booting stage and cv. DONATA vegetative growth stage), and April 9^th^ (cv. BAOXING heading stage and cv. DONATA booting stage). Mixed young leaves were sampled as biological replicates, and three biological replicates were sampled at each time point.

###

### 5.3 Transcriptome analysis

After sequencing, raw RNA reads were trimmed and mapped to the draft reference genomes by TopHat2 [[28](#_ENREF_28)] with the following parameters: --max-intron-length 500,000, --read-gap-length 10, --read-edit-dist 15, --max-insertion-length 5 and --max-deletion-length 5. The expression level (reads per kilobase of transcript per million mapped reads (RPKM) value) of each protein-coding gene was calculated by using HTSeq with default parameters [[53](#_ENREF_53)]. DESeq2 was used to normalize gene expression (BaseMean) in each sample and to identify differentially expressed genes (DEGs) with an adjusted *p*-value less than 0.05 [[54](#_ENREF_54)]. All DEGs were mapped to GO terms in the GO database (<http://www.geneontology.org/>), and significantly enriched GO terms were selected using a hyper-geometric test to develop hierarchical clusters of a sample tree by Euclidean distance. To further clarify the biological functions of DEGs, a pathway-based analysis was conducted using the KEGG database (http://www.genome.jp/kegg). Pathways with *q*-values < 0.05 were considered significantly enriched. Log2-normalized RPKM values were used to generate co-expression networks with the WGCNA package in R [[55](#_ENREF_55)]. Gene structure analysis was performed using the TAPIS pipeline [[56](#_ENREF_56)]. Mapping of high-quality PacBio reads and identification of AS events were performed by using GMAP with default settings [[56](#_ENREF_56)].

###

### 5.4 BSA-sequencing materials

To identify SNPs associated with genes involved in flowering time regulation, two pools of individuals were selected from an F_1_ mapping population of 213 individuals derived from a cross between parents with contrasting flowering times: one with an early-flowering phenotype (“Kaimo”) and one with a late-flowering phenotype (“01436”) [[20](#_ENREF_20)]. One pool was an early-flowering pool, and the other was a late-flowering pool. Four libraries were prepared (two parents and two pools) to detect SNPs associated with flowering-time candidate genes.

###

### 5.5 BSA

Sequence data of the two pools (early-flowering pool and late-flowering pool) and two parents (early-flowering phenotype: “Kaimo” and late-flowering phenotype: “01436”) were generated by the NovaSeq platform. The pipeline for trimming, read mapping, SNP/InDel calling and annotation was the same as that described above in section 4.3. Homozygous SNPs in one of the parents and heterozygous SNPs in the other parent were extracted from the ‘vcf’ output files. The homozygous SNP was used as the reference to calculate the number of reads of the genotype in the two pools. The ratio of reads harbouring the SNP that was different from the reference sequence was calculated as the SNP index of the base site. Ratios with a SNP index of the two pools less than 0.1, which might be due to sequencing or alignment errors, were filtered out. A sliding window of 1 Mb and a step size of 1 Kb were used to obtain the SNP index of the whole genome. The SNP index for each window was calculated as the average of all the SNP indices in that area of the genome. The difference in the SNP index between the two pools was calculated as the transformed Δ(SNP index).

###

### 5.6 WGCNA

A total of 8,629 DEGs between early- and late-flowering orchardgrass accessions were chosen as probes for weighted gene co-expression network (WGCN) construction, of which genes in three modules (pink, purple and green modules) were associated with vernalization (Figure S16 and 17, Table S36), including 5 CONSTANS-LIKE and 3 FT-LIKE genes. WGCN analysis (WGCNA) indicated that VRN2 is coordinated by a set of 176 genes in orchardgrass (magenta module) (Table S37). In this module, several known flowering genes were detected, including the two-component response regulators ARR9/3/1, CONSTANS/CONSTANS-LIKE, LHY and PRR37, which are involved in the circadian clock and photoperiod signalling pathways. The gibberellic acid (GA) and abscisic acid (ABA) pathway-related genes GA20ox1D, GA20ox2, PYL5 and ABI5 were also identified, which have been shown to function in flowering modulation in *A. thaliana*. Among the molecular functions, transporter activity terms and biological process: transport terms related to plant hormone transport were enriched. Remarkably, when analysing the gene expression in early- and late-flowering orchardgrass, many genes in this module showed different expression profiles after the vernalization stage. We annotated genes that were differentially expressed in the early vs late comparison, and 38 DEGs were identified (Table S38). This analysis identified a potential relationship between flowering time and several photosynthetic system- and plant transport-related genes, including those involved in photosynthesis, chlorophyll catabolic process, sodium ion transport and hormone signal transduction.

**Supplementary Figure Legends**

**Figure S1** The orchardgrass genome landscape. Track a represents the seven chromosomes on a Mb scale. Track b represents the chromosomal distribution of 603 orthologues of *A. thaliana* flowering genes. Track c represents the chromosomal distribution of gene models that were annotated, where gene density ranged from 371 bp/Mb to 380,434 bp/Mb. Track d represents the chromosomal distribution of repeat sequences, where the density of the repeat sequences was 74,261 bp/Mb to 903,485 bp/Mb. Track e represents the chromosomal distribution of TE density, and the TE density axis range was 74,261 bp/Mb–895,797 bp/Mb. Track f represents the GC content along the assembled genome, which ranged between 41.0989%/Mb and 48.0363%/Mb. Track g represents the pooled gene expression level of five tissues (root, stem, leaf, flower, and spike). Track h represents the chromosomal distribution of SNPs identified from 54 wild germplasm accessions, 11 cultivars and 11 unknown orchardgrass collection samples. The range of SNP density was 3,034/Mb–111,599/Mb.

**Figure S2** Workflow of the orchardgrass genome assembly.

**Figure S3** K-mer frequency distributions in orchardgrass. The x axis represents the sequence depth, and the y axis represents the frequency of the K-mer.

**Figure S4** Scaffold Hi-C contact map data analysis. The x and y axes indicate the mapping positions of the first and second read in the read pair, respectively, grouped into bins. The colour of each square indicates the number of read pairs within that bin. White vertical and black horizontal lines have been added to show the borders between scaffolds. Scaffolds less than 1 Mb are excluded.

**Figure S5** The chromosome number of diploid orchardgrass (genotype 2006-1).

**Figure S6** Consistency between the Hi-C and BioNano results. Blue dots indicate the negative direction, and red dots indicate the positive direction.

**Figure S7** The density of TEs surrounding genes. The density was determined as the number of genes inserted by TEs into one locus to all genes inserted by TEs. TSS represents the transcript start point, and TTS represents the transcript end point. Dgl represents orchardgrass, Ata represents *A. tauschii*, Bdi represents *B. distachyon*, and Tur represents *T. urartu*.

**Figure S8** The distribution of divergence time for LTRs/Gypsy and LTRs/Copia.

**Figure S9** Synteny analysis of seven chromosomes from orchardgrass (Dgl) to twelve chromosomes from *O. sativa* (Osa) and seven chromosomes from *A. tauschii* (Ata).

**Figure S10** REM family in orchardgrass. (a) Expression levels of REM genes in different tissues. (b) Expression levels of REM genes in five flowering stages (BV, before vernalization; VE, vernalization; AV, after vernalization; VG, vegetative growth; BH, before heading; and HT, heading stage). The y axis represents relative expression, and the x axis represents the different stages. BX indicates the orchardgrass cultivar BAOXING, and DON indicates the orchardgrass cultivar DONATA. (c) Expansion time of REM genes. (d) The density of transposons around REMs and all genes in orchardgrass. The density was determined as the number of genes inserted by TEs into one locus to all genes inserted by TEs. TSS represents the transcript start point, and TTS represents the transcript end point.

**Figure S11** Phylogenetic tree of 76 orchardgrass accessions. The colours of the inner circle indicate the materials from different regions: blue, Eastern Mediterranean; yellow, Central Asia; pink, East Asia; green, Western Mediterranean; and orange, Northern Europe. The colours of the outer circle indicate the wild (red), cultivar (light blue) and uncertain (dark blue) orchardgrass accessions.

**Figure S12** Structure analysis of 76 orchardgrass accessions with different K values.

**Figure S13** PCA plot of the first two components (PC1 and PC2) of 43 autotetraploid orchardgrass accessions.

**Figure S14** Phylogenetic tree of 43 autotetraploid orchardgrass accessions. The colours of the inner circle indicate the materials from different regions: blue, Eastern Mediterranean; yellow, Central Asia; pink, East Asia; green, Western Mediterranean; and red, Northern Europe. The colours of the outer circle indicate the wild (dark green) and cultivar (light green) orchardgrass accessions.

**Figure S15** Analysis of important flowering-related orthologues in orchardgrass. (a) Expression levels of several key orthologues associated with flowering time (BV, before vernalization; VE, vernalization; AV, after vernalization; VG, vegetative growth; BH, before heading; and HT, heading stage). (b) Phylogenetic tree of FT orthologues in orchardgrass, rice, *B. distachyon*, *H. vulgare*, *A. tauschii*, and *T. urartu*. The red line indicates five FT orthologues that underwent expansion during orchardgrass evolution.

**Figure S16** Nucleotide diversity (π) estimated in wild (red) and cultivated (green) orchardgrass (a) and the *FST* value (b) and patterns of LD in cultivated (c) and wild (d) orchardgrass in the 4.426-Mb region of orchardgrass chromosome 06.

**Figure S17** Comparison of *AGL61* expression during the five developmental stages

in orchardgrass. Error bars indicate ±s.d.; n = 3 biological replicates.

**Figure S18** Module-sample relationship. The heatmap shows the correlation between modules and samples, and deeper red indicates a stronger correlation.

**Figure S19** Expression pattern of genes in green, pink and purple modules. (a) and (b) indicate the genes of the green module for the early- and late-flowering phenotypes, (c) and (d) indicate the genes of the pink module for the early-and late-flowering phenotypes, and (e) and (f) indicate the genes of the purple module for the early- and late-flowering phenotypes.

**References**

1. Sharawy SM. Karyotype Analysis and Systematic Relationships in the Egyptian Astragalus L. (Fabaceae). International Journal of Botany. 2007;22(2):125-7.

2. Bo W, Elizabeth T, Michael R, A CT, Ting H, Yinping J, et al. Unveiling the complexity of the maize transcriptome by single-molecule long-read sequencing. Nature Communications. 2016;7:11708.

3. Rhoads A, Au KF. PacBio Sequencing and Its Applications. Genomics,Proteomics & Bioinformatics. 2015;13(5):278-89.

4. Liu B, Shi Y, Yuan J, Hu X, Zhang H, Li N, et al. Estimation of genomic characteristics by analyzing k-mer frequency in de novo genome projects. Quantitative Biology. 2013;35(s 1–3):62-7.

5. Zhang Q, Chen W, Sun L, Zhao F, Huang B, Yang W, et al. The genome of Prunus mume. Nature Communications. 2011;3(4):187-90.

6. Doležel J, Greilhuber J, Suda J. Estimation of nuclear DNA content in plants using flow cytometry. Nature Protocols. 2007;2(9):2233-44.

7. Chin CS, Peluso P, Sedlazeck FJ, Nattestad M, Concepcion GT, Clum A, et al. Phased Diploid Genome Assembly with Single Molecule Real-Time Sequencing. Nature Methods. 2016;13(12):1050-4.

8. Chin CS, Alexander DH, Marks P, Klammer AA, Drake J, Heiner C, et al. Nonhybrid, finished microbial genome assemblies from long-read SMRT sequencing data. Nature Methods. 2013;10(6):563.

9. Li H. Toward better understanding of artifacts in variant calling from high-coverage samples. Bioinformatics. 2014;30(20):2843-51.

10. A A, JO K, JN B, R D, A K, L C, et al. In vitro, long-range sequence information for de novo genome assembly via transposase contiguity. Genome Research. 2014;24(12):2041-9.

11. Lieberman-Aiden E, Dekker J. Comprehensive Mapping of Long-Range Interactions Reveals Folding Principles of the Human Genome. Science. 2009;326(5950):289.

12. Putnam NH, O'Connell BL, Stites JC, Rice BJ, Blanchette M, Calef R, et al. Chromosome-scale shotgun assembly using an in vitro method for long-range linkage. Genome Research. 2016;26(3):342.

13. Zaharia M, Bolosky WJ, Curtis K, Fox A, Patterson D, Shenker S, et al. Faster and More Accurate Sequence Alignment with SNAP. Corr. 2011:2011.

14. English AC, Salerno WJ, Reid JG. PBHoney: identifying genomic variants via long-read discordance and interrupted mapping. Bmc Bioinformatics. 2014;15(1):180.

15. Walker BJ, Abeel T, Shea T, Priest M, Abouelliel A, Sakthikumar S, et al. Pilon: An Integrated Tool for Comprehensive Microbial Variant Detection and Genome Assembly Improvement. Plos One. 2014;9(11):e112963.

16. Parra G, Bradnam K, Korf I. CEGMA: a pipeline to accurately annotate core genes in eukaryotic genomes. Bioinformatics. 2007;23(9):1061-7.

17. Simão FA, Waterhouse RM, Ioannidis P, Kriventseva EV, Zdobnov EM. BUSCO: assessing genome assembly and annotation completeness with single-copy orthologs. Bioinformatics. 2017;31(19):3210-2.

18. Li H, Handsaker B, Wysoker A, Fennell T, Ruan J, Homer N, et al. The Sequence Alignment/Map format and SAMtools. Bioinformatics. 2009;25(16):2078-9.

19. Bushman BS, Larson SR, Tuna M, West MS, Hernandez AG, Vullaganti D, et al. Orchardgrass ( Dactylis glomerata L.) EST and SSR marker development, annotation, and transferability. Tagtheoretical & Applied Geneticstheoretische Und Angewandte Genetik. 2011;123(1):119-29.

20. Zhao X, Huang L, Zhang X, Wang J, Yan D, Li J, et al. Construction of high-density genetic linkage map and identification of flowering-time QTLs in orchardgrass using SSRs and SLAF-seq. Scientific reports. 2016;6:29345.

21. Smit A, Hubley R. RepeatModeler Open-1.0. Available fom <http://www> repeatmasker org. 2008.

22. Xu Z, Wang H. LTR_FINDER: an efficient tool for the prediction of full-length LTR retrotransposons. Nucleic acids research. 2007;35(suppl_2):W265-W8.

23. Price AL, Jones NC, Pevzner PA. De novo identification of repeat families in large genomes. Bioinformatics. 2005;21(suppl_1):i351-i8.

24. Chen N. Using RepeatMasker to identify repetitive elements in genomic sequences. Current protocols in bioinformatics. 2004;5(1):4.10. 1-4.. 4.

25. SF A, W G, W M, EW M, DJ L. Basic local alignment search tool. Journal of Molecular Biology. 1990;215(3):403-10.

26. Yu XJ, Zheng HK, Wang J, Wang W, Su B. Detecting lineage-specific adaptive evolution of brain-expressed genes in human using rhesus macaque as outgroup. Genomics. 2006;88(6):745-51.

27. Cook CE, Bergman MT, Cochrane G, Apweiler R, Birney E. The European Bioinformatics Institute in 2017: data coordination and integration. Nucleic Acids Research. 2018;46(D1):D21.

28. Kim D, Pertea G, Trapnell C, Pimentel H, Kelley R, Salzberg SL. TopHat2: accurate alignment of transcriptomes in the presence of insertions, deletions and gene fusions. Genome biology. 2013;14(4):R36.

29. Ghosh S, Chan CK. Analysis of RNA-Seq Data Using TopHat and Cufflinks: Springer New York; 2016. 339-61 p.

30. Haas BJ, Delcher AL, Mount SM, Wortman JR, Jr SR, Hannick LI, et al. Improving the Arabidopsis genome annotation using maximal transcript alignment assemblies. Nucleic Acids Research. 2003;31(19):5654-66.

31. Keller O, Kollmar M, Stanke M, Waack S. A novel hybrid gene prediction method employing protein multiple sequence alignments. Bioinformatics. 2011;27(6):757-63.

32. Burge CB, Karlin S. Finding the genes in genomic DNA. Current Opinion in Structural Biology. 1998;8(3):346-54.

33. Majoros WH, Pertea M, Salzberg SL. TigrScan and GlimmerHMM: two open source ab initio eukaryotic gene-finders. Bioinformatics. 2004;20(16):2878-9.

34. Blanco E, Parra G, Guigó R. Using geneid to Identify Genes. Current Protocols in Bioinformatics. 2007;18(1):Unit 4.3.

35. Haas BJ, Salzberg SL, Wei Z, Pertea M, Allen JE, Orvis J, et al. Automated eukaryotic gene structure annotation using EVidenceModeler and the Program to Assemble Spliced Alignments. Genome Biology. 2008;9(1):R7.

36. Altschul SF, Madden TL, Schäffer AA, Zhang J, Zhang Z, Miller W, et al. Gapped BLAST and PSI-BLAST: a new generation of protein database search programs. 1997.

37. Finn RD, Attwood TK, Babbitt PC, Bateman A, Bork P, Bridge AJ, et al. InterPro in 2017—beyond protein family and domain annotations. Nucleic Acids Research. 2017;45(Database issue):D190-D9.

38. Finn RD, Mistry J, Tate J, Coggill P, Heger A, Pollington JE, et al. Pfam protein families database. Nucleic Acids Research. 2010.

39. Zdobnov EM, Apweiler R. InterProScan – an integration platform for the signature-recognition methods in InterPro. Bioinformatics. 2001;17(9):847-8.

40. Finn RD, Clements J, Arndt W, Miller BL, Wheeler TJ, Schreiber F, et al. HMMER web server: 2015 update. Nucleic Acids Research. 2015;43(1):30-8.

41. Rogers MF, Thomas J, Reddy AS, Ben-Hur A. SpliceGrapher: detecting patterns of alternative splicing from RNA-Seq data in the context of gene models and EST data. Genome biology. 2012;13(1):R4.

42. Edgar RC. MUSCLE: multiple sequence alignment with high accuracy and high throughput. Nucleic Acids Research. 2004;32(5):1792-7.

43. Stamatakis A. RAxML version 8: a tool for phylogenetic analysis and post-analysis of large phylogenies. Bioinformatics. 2014;30(9):1312-3.

44. Yang Z. Yang ZH.. PAML 4: Phylogenetic analysis by maximum likelihood. Mol Biol Evol 24: 1586-1591. 2007;24(8):1586-91.

45. Han MV, Thomas GWC, Lugomartinez J, Hahn MW. Estimating Gene Gain and Loss Rates in the Presence of Error in Genome Assembly and Annotation Using CAFE 3. Molecular Biology & Evolution. 2013;30(8):1987-97.

46. Tang H, Bowers JE, Wang X, Ming R, Alam M, Paterson AH. Synteny and Collinearity in Plant Genomes. Science. 2008;320(5875):486-8.

47. Li H, Durbin R. Fast and accurate short read alignment with Burrows–Wheeler transform. bioinformatics. 2009;25(14):1754-60.

48. Vilella AJ, Severin J, Ureta-Vidal A, Heng L, Durbin R, Birney E. EnsemblCompara GeneTrees: Complete, duplication-aware phylogenetic trees in vertebrates. Genome Research. 2009;19(2):327-35.

49. Yang J, Lee SH, Goddard ME, Visscher PM. GCTA: a tool for genome-wide complex trait analysis. American Journal of Human Genetics. 2011;88(1):76-82.

50. Alexander DH, Novembre J, Lange K. Fast model-based estimation of ancestry in unrelated individuals. Genome Research. 2009;19(9):1655-64.

51. Bouché F, Lobet G, Tocquin P, Périlleux C. FLOR-ID: an interactive database of flowering-time gene networks in Arabidopsis thaliana. Nucleic Acids Research. 2015;44(Database issue):D1167-D71.

52. Li L, Stoeckert CJ, Roos DS. OrthoMCL: identification of ortholog groups for eukaryotic genomes. Genome Research. 2003;13(9):2178-89.

53. Anders S, Pyl PT, Huber W. HTSeq—a Python framework to work with high-throughput sequencing data. Bioinformatics. 2015;31(2):166-9.

54. Anders S, Huber W. Differential expression analysis for sequence count data. Genome biology. 2010;11(10):R106.

55. Langfelder P, Horvath S. Langfelder P, Horvath S. WGCNA: an R package for weighted correlation network analysis. BMC Bioinform 9: 559. Bmc Bioinformatics. 2008;9(1):559.

56. Abdelghany SE, Hamilton M, Jacobi JL, Ngam P, Devitt N, Schilkey F, et al. A survey of the sorghum transcriptome using single-molecule long reads. Nature Communications. 2016;7:11706.

57. Zhao X, Huang L, Zhang X, Wang J, Yan D, Ji L, et al. Construction of high-density genetic linkage map and identification of flowering-time QTLs in orchardgrass using SSRs and SLAF-seq. Scientific Reports. 2016;6:29345.
